# Type VI secretion system-mediated bacterial antagonism in the classroom

**DOI:** 10.1099/acmi.0.001128.v3

**Published:** 2026-06-12

**Authors:** Alejandro Tejada-Arranz, Peter Ringel, Lin Lin, Marek Basler

**Affiliations:** 1 Biozentrum, University of Basel, Basel, Switzerland

**Keywords:** cell–cell interactions, live-cell imaging, type VI secretion system (T6SS)

## Abstract

The study of bacterial antagonism, and specifically type VI secretion system (T6SS)-mediated antagonism, has gained momentum over the last decade in research laboratories across the world. The T6SS is a molecular machine that mediates interbacterial antagonism, as well as interactions with the host in the case of pathogenic or commensal organisms. Here, we describe a set of protocols and materials that can be used as resources to develop practical courses to study the assembly and firing of this machinery. These resources help train students on basic molecular biology techniques and secretion and competition assays, as well as fluorescence microscopy. The full set of activities is appropriate for students at the level of second- to third-year students in the Bachelor of Biology and can be carried out in 4 days requiring minimal specialized equipment. This course allows the students to familiarize themselves with standard microbiology laboratory approaches and learn how to study the function of a complex molecular machinery.

## Data Summary

DNA sequences and supplementary videos are available on https://zenodo.org/records/20155056.

## Introduction

The type VI secretion system (T6SS) is a molecular machine that mediates interbacterial antagonism, as well as interactions with the host in the case of pathogenic or commensal organisms [1,2]. The T6SS assembles into a needle-like machinery inside the bacterial cytoplasm composed of an inner tube consisting of the Hcp protein surrounded by a sheath structure formed by TssB and [3]. The tip of the needle, generally constituted by VgrG and PAAR proteins, is loaded with effectors that vary widely between species and even strains. The contraction of the sheath pushes the needle structure through the membranes of the aggressor bacterium, with enough power to also pierce the cell envelope of neighbouring cells. The effectors are released into the surrounding environment, i.e. the extracellular milieu or the cytoplasm or periplasm of a neighbouring bacterium. The targeted bacterium will often die as a consequence of such attacks unless it possesses specific defence mechanisms against the injected effectors [4]. For instance, sister cells from the same species will possess cognate immunity proteins that are encoded next to the effectors and protect against their effect [5].

For our practical courses, we have constructed a set of strains and designed protocols that facilitate the study of several aspects of the dynamics of this machinery, including (i) fluorescently labelled strains that enable the study of the assembly and firing of this machinery by microscopy and (ii) knockout strains in different structural, regulatory or effector components of this machinery, which allow the development of strategies to knockout genes and study their phenotypes through fluorescence microscopy and bacterial competition assays. The required materials are low-cost and readily available in molecular biology teaching laboratories. We utilize a bacterium, *Acinetobacter baylyi* isolate BD413 (ATCC 33305). This strain encodes a T6SS [6, 7], is non-pathogenic, naturally transformable [8] and is classified as a biosafety level 1 organism, thus requiring only minimal safety precautions and protocols. To study the effects of the T6SS of *A. baylyi*, we also use an *Escherichia coli* MG1655 strain that is LacZ+ and gentamicin-resistant as prey strain [6].

There are a number of literature resources with ideas for practical microbiology courses at the bachelor level. Most of these resources focus on single techniques or are geared towards species identification in an environmental or clinical setting [9,13]. Our course familiarizes the students with a full molecular microbiology workflow in 4 days: from primer design and mutant construction to mutant screening, functional assays and hypothesis generation. To the best of our knowledge, there are no available resources of this kind, where the students learn about basic microbiology and molecular biology techniques, approaches to study bacterial antagonism through competition assays, fluorescence microscopy, secretion assays and protein bioinformatics.

In this resource paper, we include the teaching materials that we have developed and adjusted over 5+ consecutive iterations of our yearly 3–4-day-long practical microbiology teaching course, making it a valuable contribution to the microbiology teaching community.

## Target level and learning goals

The presented activities require a basic understanding of molecular biology and microbiology approaches, DNA and protein composition, the genetic code, antibiotic resistance markers and bacterial culture. The activities were designed for students at the bachelor’s level and were tested on third-year BSc Biology students at the University of Basel, Switzerland. For an example theoretical background for students, we refer to sections 2 and 3 of the Supplementary Material containing a sample student script.

**Fig. 1.**
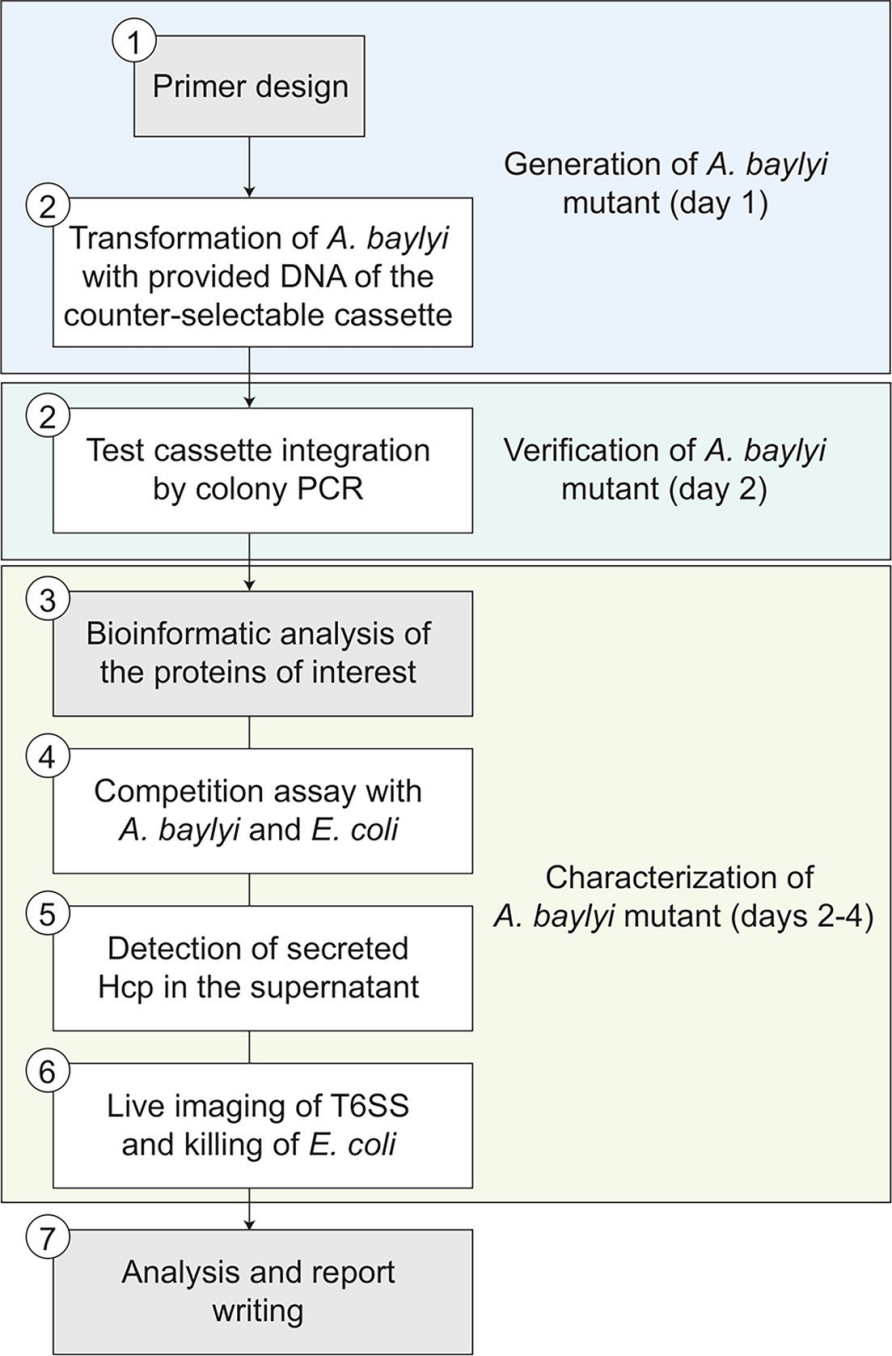
Workflow overview. Shaded in grey are the activities that need to be carried out *in silico*.

We divided the work into seven activities, summarized in Fig. 1, that can be carried out over 4 days and that target different learning competences:

- Activity 1: Primer design for the construction of knockout strains of T6SS-related genes of *A. baylyi* ADP1.
- Activity 2: Construction of a knockout strain of *A. baylyi*. The activity involves PCR amplification, transformation and selection of *A. baylyi* and screening for clones of interest.
- Activity 3: Bioinformatic analysis of proteins of interest. What can we learn from the sequences of these proteins by using freely available prediction tools?
- Activity 4: Competition assay with *A. baylyi* and *E. coli*. The activity involves mixing, diluting and plating bacteria on media with different compositions to perform both qualitative and quantitative killing assays to understand if and how killing of the mutants of interest is impacted compared to the parental strain.
- Activity 5: Detection of Hcp secretion in the supernatant of *A. baylyi* cultures. This includes precipitating proteins from the supernatant of *A. baylyi* cultures, as well as preparing and running an SDS-PAGE of the precipitate to detect the presence of Hcp. It is important to note that this activity requires the utilization of dangerous chemicals [trichloroacetic acid (TCA), acetone, acrylamide, N,N,N’,N’-Tetramethyl ethylenediamine (TEMED), ammonium persulfate (APS) and dithiothreitol (DTT)], and it must not be performed in the absence of appropriate protective equipment.
- Activity 6: Live imaging of T6SS dynamics and *E. coli* killing by fluorescence microscopy. This activity involves the preparation of agar pads for immobilization of fluorescent bacteria and a fluorescent microscope. Students prepare the samples and are assisted by trained supervisors in the imaging of the samples.
- Activity 7: Report writing exercise. Students organize their findings and write a short coherent report in the style of a scientific publication, referring to the script as bibliography and in the materials and methods section.

## Technical prerequisites

Each activity requires access to different materials:

- Activity 1: primer design tools. We use the freely accessible SnapGene Viewer and provide sequence files with the genomic regions of interest (available on Zenodo https://doi.org/10.5281/zenodo.20155056).
- Activity 2: we need basic molecular biology equipment, including thermocyclers, PCR reagents, agarose gel electrophoresis equipment and a UV transilluminator. A fluorescently labelled parental strain of *A. baylyi* is used, and lysogeny broth (LB) plates with 50 µg ml^−1^ kanamycin are needed to select for *A. baylyi* transformants. The primers are indicated in Table S1 (available in the online Supplementary Material).
- Activity 3: we use protein sequences that can be obtained from the DNA files above and freely accessible online tools for prediction of protein structure/properties.
- Activity 4: this activity requires four types of plates:
  - Qualitative killing assays: LB agar plates with 20 µg ml^−1^ CPRG (chlorophenol red-*β*-d-galactopyranoside) and 0.1 mM IPTG (isopropyl-*β*-d-1-thiogalactopyranoside) and LB agar plates with 40 µg ml^−1^ 5-bromo-4-chloro-3-indolyl-*β*-d-galactopyranoside and 0.1 mM IPTG.
  - Quantitative killing assays: LB agar plates (without additives) for competition, LB agar plates with 100 µg ml^−1^ streptomycin to select for *A. baylyi* and LB agar plates with 20 µg ml^−1^ gentamicin to select for *E. coli*.

- Activity 5: this activity requires TCA for the precipitation, acetone for washes and standard reagents and equipment for SDS-PAGE and gel staining.
- Activity 6: this activity requires basic fluorescence microscopy equipment, including glass slides and coverslips, agarose-LB pads, propidium iodide and a fluorescence microscope with a 100× objective, a phase contrast ring, a camera for time-lapse microscopy, appropriate excitation sources and filters to detect fluorescence emitted by monomeric superfolder GFP (msfGFP) and mCherry2. For image analysis, we use the freely available Fiji (Fiji is Just ImageJ) [14] software.
- Activity 7: standard text editor and spreadsheet software are sufficient.

## Provided bacterial strains

Activities 1 and 3 are performed *in silico* and require no bacterial cultures. For activities 2 and 4–6, the bacterial strains indicated in Table 1 are provided. Each student or student group receives one mutant of a component of a T6SS that they need to study to understand the role of this gene for T6SS assembly, firing or killing activity.

**Table 1.** List of strains needed for the proposed work

Strain	Activity	Antibiotic resistance marker	Fluorescent labels	Remarks
*A. baylyi* ADP1 StrepR	2, 4, 5	*rpsL* K88R, streptomycin resistant	TssB-msfGFP ClpV-mCherry2	Parental strain for chromosomal knockout construction; provided negative control for colony PCR and positive control for killing assays and microscopy; deposited at the DSMZ collection under number DSM 122038.
*A. baylyi* ADP1 Δ*aciad2685::KanR-rpsL^WT^*	2	KanR, sensitive to streptomycin	TssB-msfGFP ClpV-mCherry2	Provided positive control for colony PCR; knockout of *aciad2685*, *tagY*, a protein of unknown function important for T6SS assembly; deposited at the DSMZ collection under number DSM 122039.
*A. baylyi* ADP1 Δ*aciad2689::KanR-rpsL^WT^*	2	KanR, sensitive to streptomycin	TssB-msfGFP ClpV-mCherry2	Provided positive control for colony PCR; knockout of *aciad2689*, *hcp*, structural component of the T6SS; deposited at the DSMZ collection under number DSM 122040.
*A. baylyi* ADP1 Δ*aciad2693::KanR-rpsL^WT^*	2	KanR, sensitive to streptomycin	TssB-msfGFP ClpV-mCherry2	Provided positive control for colony PCR; knockout of *aciad2693*, *tslA*, involved in contact-dependent T6SS assembly; deposited at the DSMZ collection under number DSM 1220341.
*A. baylyi* ADP1 Δ*aciad2699::KanR-rpsL^WT^*	2	KanR, sensitive to streptomycin	TssB-msfGFP ClpV-mCherry2	Provided positive control for colony PCR; knockout of *aciad2699*, *tagX*, a carboxypeptidase important for T6SS assembly; ; deposited at the DSMZ collection under number DSM 122042.
*A. baylyi* ADP1 Δ*aciad3425::KanR-rpsL^WT^*	2	KanR, sensitive to streptomycin	TssB-msfGFP ClpV-mCherry2	Provided positive control for colony PCR; knockout of *aciad3425*, Tle1, a lipase T6SS effector; deposited at the DSMZ collection under number DSM 122043.
*A. baylyi* ADP1 Δ*aciad2685*	4, 5, 6	*rpsL* K88R, streptomycin resistant	TssB-msfGFP ClpV-mCherry2	Provided mutant strain for killing assays, Hcp secretion detection and microscopy; clean knockout of *aciad2685*, *tagY*, a protein of unknown function important for T6SS assembly; deposited at the DSMZ collection under number DSM 122044.
*A. baylyi* ADP1 Δ*aciad2689*	4, 5, 6	*rpsL* K88R, streptomycin resistant	TssB-msfGFP ClpV-mCherry2	Provided mutant strain for killing assays, Hcp secretion detection and microscopy; clean knockout of *aciad2689*, *hcp*, structural component of the T6SS; deposited at the DSMZ collection under number DSM 122045.
*A. baylyi* ADP1 Δ*aciad2693*	4, 5, 6	*rpsL* K88R, streptomycin resistant	TssB-msfGFP ClpV-mCherry2	Provided mutant strain for killing assays, Hcp secretion detection and microscopy; clean knockout of *aciad2693*, *tslA*, involved in contact-dependent T6SS assembly; deposited at the DSMZ collection under number DSM 122046.
*A. baylyi* ADP1 Δ*aciad2699*	4, 5, 6	*rpsL* K88R, streptomycin resistant	TssB-msfGFP ClpV-mCherry2	Provided mutant strain for killing assays, Hcp secretion detection and microscopy; clean knockout of *aciad2699*, *tagX*, a carboxypeptidase important for T6SS assembly; deposited at the DSMZ collection under number DSM 122047.
*A. baylyi* ADP1 Δ*aciad3425*	4, 5, 6	*rpsL* K88R, streptomycin resistant	TssB-msfGFP ClpV-mCherry2	Provided mutant strain for killing assays, Hcp secretion detection and microscopy; clean knockout of *aciad3425*, Tle1, a lipase T6SS effector; deposited at the DSMZ collection under number DSM 122048.

KanR, kanamycin resistant.

## Procedure

The full protocols and the script are available in File S1, including teaching notes. The full set of activities, including lectures and assisted report writing, can be completed in 4 days. A sample schedule can be found in Table 2. Example results that can be obtained in the different experiments are provided in File S2. Microscopy videos are provided as Videos S1–S9 (available on Zenodo: https://doi.org/10.5281/zenodo.20155056).

**Table 2.** Sample schedule of the proposed activities. In blue, experimental sections that need to be carried out in the research lab. In yellow, theoretical/analysis sections that are carried out in a classical classroom or in a computer classroom

## Troubleshooting

In Table 3, we have collected a list of some frequent issues and their possible causes/solutions.

**Table 3.** Troubleshooting guide

Experiment	Issue	Cause	Solution
3	No bands in the gel	Some reagent is missing	Repeat PCR
		Too much starting material/LB agar bits in the reaction mix	Add less bacteria, being careful not to poke the agar
		Wrong PCR settings	Try reducing the annealing temperature and checking that the elongation time is sufficient
	Multiple bands in the gel	Unspecific primer binding	Increase the annealing temperature.
4	Variable replicates	Insufficient mixing of the strains	Make sure to mix the strains well before plating
	There are colonies in all dilutions	Contaminated medium	Pay attention to working in aseptic conditions without contaminating the LB medium.
	The spots are too red/blue or not red/blue enough	Too much/too little incubation time	Incubation time is an important variable of these qualitative killing assays. Make sure to take multiple pictures over time to make sure of capturing the most information.
5	Upon addition of loading buffer to the protein sample, the sample turns yellow, and the proteins migrate poorly during electrophoresis	Residual TCA (strong acid)	Make sure to completely remove the supernatant and wash the pellet effectively with acetone. If, despite this, the buffer colour still changes, add 1M Tris-HCl (pH 8.8), 1 µl at a time, until the sample turns back to blue colour.
	Hcp band ran out of the bottom of the gel	Incorrect gel percentage	Ensure a 12–15% acrylamide in the SDS-PAGE gel.
	Negative control shows a ladder-like pattern similar to the whole cell lysate.	Cell lysis due to rough resuspension handling/vortexing or due to harvest of cells entering death phase	Handle the sample gently and ensure harvesting the culture at the correct OD (<1.4) to avoid excessive cell lysis. Only few bands are expected.
	No bands are observed	Poor/old culture or suboptimal TCA precipitation	Make sure to use a fresh mid-exponential phase culture and to wash the cells. Observing a few bands would suggest that the precipitation worked correctly.
6	No assembly dynamics	*A. baylyi* T6SS dynamics are lost during incubation on agar pads – with fluorescent aggregates increasing over time	Put *A. baylyi* on the agar pad immediately before imaging; image close to the pad edge to avoid potential effects of lack of oxygen on T6SS dynamics.
	Bacteria are floating around	There was too much liquid on the pad at the time of imaging	Make sure to pipette only small volumes onto the pad, and wait a few seconds for the liquid to be absorbed before putting on the coverslip.

## Limitations

Even though this practical module was highly appreciated by the students, we lack ethical approval (informed consent) to release the results of past surveys. Nevertheless, we believe that this resource will be valuable for the microbiology teaching community to design other practical courses that may include the strains and some of the assays described here.

## Supplementary material

10.1099/acmi.0.001128.v3 Supplementary Material 1.

10.1099/acmi.0.001128.v3 Uncited Supplementary Material 2.

10.1099/acmi.0.001128.v3 Uncited Supplementary Material 3.

## Abbreviations

APS: ammonium persulfate
CPRG: chlorophenol red-*β*-d-galactopyranoside
IPTG: isopropyl-*β*-d-1-thiogalactopyranoside
KanR: Kanamycin resistant
LB: lysogeny broth
msfGFP: monomeric superfolder GFP
TCA: trichloroacetic acid
TEMED: N,N,N’,N’-Tetramethyl ethylenediamine
T6SS: type VI secretion system
