# Supplementary material for: Type VI secretion system-mediated bacterial antagonism in the classroom
Source: Access Microbiol. 2026 Jun 12;8(6):001128.v3. doi: 10.1099/acmi.0.001128.v3 (PMC13274762; doi:10.1099/acmi.0.001128.v3)
Supplement: Supplementary Material 1. [file acmi-8-01128-s001.pdf]

Functional Characterization of Type VI Secretion System Components in *Acinetobacter baylyi*

Table of contents

1 Preface ... 1

2 Lecture 1: Introduction to the T6SS and *Acinetobacter baylyi* ... 3

2.1 Introduction to the T6SS ... 3

2.2 Introduction to *Acinetobacter baylyi* ... 4

3 Lecture 2: Mutagenesis in *A. baylyi* ... 6

3.1. Principles of mutagenesis in *A. baylyi* ... 6

3.2. Principle of overlap extension PCR (OE-PCR) ... 7

4. Experiment 1: Primer design ... 8

4.1. Theory ... 8

4.2. Primer design protocol ... 11

5. Experiment 2: Protein bioinformatics ... 16

6. Experiment 3: Chromosomal knockout in *A. baylyi* ... 19

6.1. Transformation of *A. baylyi* ADP1 with counter-selectable cassette by PCR ... 19

6.1.1. Materials ... 19

6.1.2. Reagents ... 19

6.1.3. Method ... 19

6.2. Colony PCR ... 20

6.2.1. Materials ... 22

6.2.2. Reagents ... 22

6.2.3. Method ... 22

6.3. DNA gel ... 23

6.3.1. Materials ... 23

6.3.2. Reagents ... 24

6.3.3. Method ... 24

7. Experiment 4: Bacterial competition assay ... 25

7.1. Theory ... 25
7.2. Protocol ... 28
7.2.1. Materials ... 28
7.2.2. Reagents ... 28
7.2.3. Method ... 28
8. Experiment 5: Detection of Hcp secretion in the supernatant of *A. baylyi* ADP1 cultures ... 31
3.1 Materials ... 31
3.2 Reagents ... 31
3.3 Method ... 32
3.3.1 SDS PAGE Gel ... 32
3.3.2 Hcp detection ... 33
9. Experiment 6: Imaging of T6SS in *A. baylyi* ... 35
9.1. Materials ... 35
9.2. Reagents ... 35
9.3. Method ... 36
9.3.1. Killing of *E. coli* by *A. baylyi* ... 36
9.3.2. T6SS assemblies in *A. baylyi* ... 37
9.4. Image Analysis with ImageJ/Fiji ... 37
9.4.1. Editing images in ImageJ/Fiji ... 37
9.4.2. Analysis of T6SS dynamics in *A. baylyi* ... 43
9.4.3. Analysis of *E. coli* killing by *A. baylyi* ... 43
9.4.4. Analysis of single effector *A. baylyi* strains ... 43
10. Appendix ... 45
10.1. Bacterial strains ... 45
10.2. Safety ... 46
11. References ... 53

1 Preface

In this practical course, you will study a dynamic molecular nanomachine, the Type VI Secretion System (T6SS), which is used by the model organism *Acinetobacter baylyi* ADP1 to kill competing bacteria such as *Escherichia coli*. Read the introduction to learn more about the T6SS and *A. baylyi*. **Your goal is to discover the roles of various T6SS genes in assembly and function of this secretion system**. To achieve this goal in a short time, you will take advantage of the fact that *A. baylyi* is a naturally competent bacterium that actively takes up DNA from its environment and inserts it into its chromosome by homologous recombination. In addition, certain experiments that are usually performed sequentially will be performed in parallel using previously prepared materials and strains. However, you will learn and practice all steps necessary to perform this kind of research.

The basis for uncovering roles of genes in our bacteria is the generation of gene mutations followed by qualitative and quantitative comparisons of the generated mutant and the parental wild-type phenotypes. On **Day 1 and 2**, you will learn how to perform targeted, two-step mutagenesis of a gene of interest in *A. baylyi* and how to isolate such mutants. You will design your own PCR primers for such mutagenesis. You will amplify and check (by colony PCR) that the provided DNA fragments have been successfully introduced in the parental strain of *A. baylyi*. You will learn how to predict protein functions using bioinformatics tools, as well as how to analyze fluorescence microscopy images and how to perform quantitative and qualitative bacterial killing assays. On **Day 3**, you will perform these bacterial killing assays, you will detect secreted components in the supernatant and you will image your own strains in the microscope. You will compare three T6SS-related properties of your *A. baylyi* mutant to those of the wild-type: (i) killing and lysis of competing bacteria by competition on plates and microscopy, (ii) secretion of the tube component Hcp and (iii) assembly of T6SSs in living bacterial cells under the microscope. On **Day 4**, you will analyze your results and propose a hypothesis explaining the role of the mutated gene in T6SS function, and you will present some of your results.

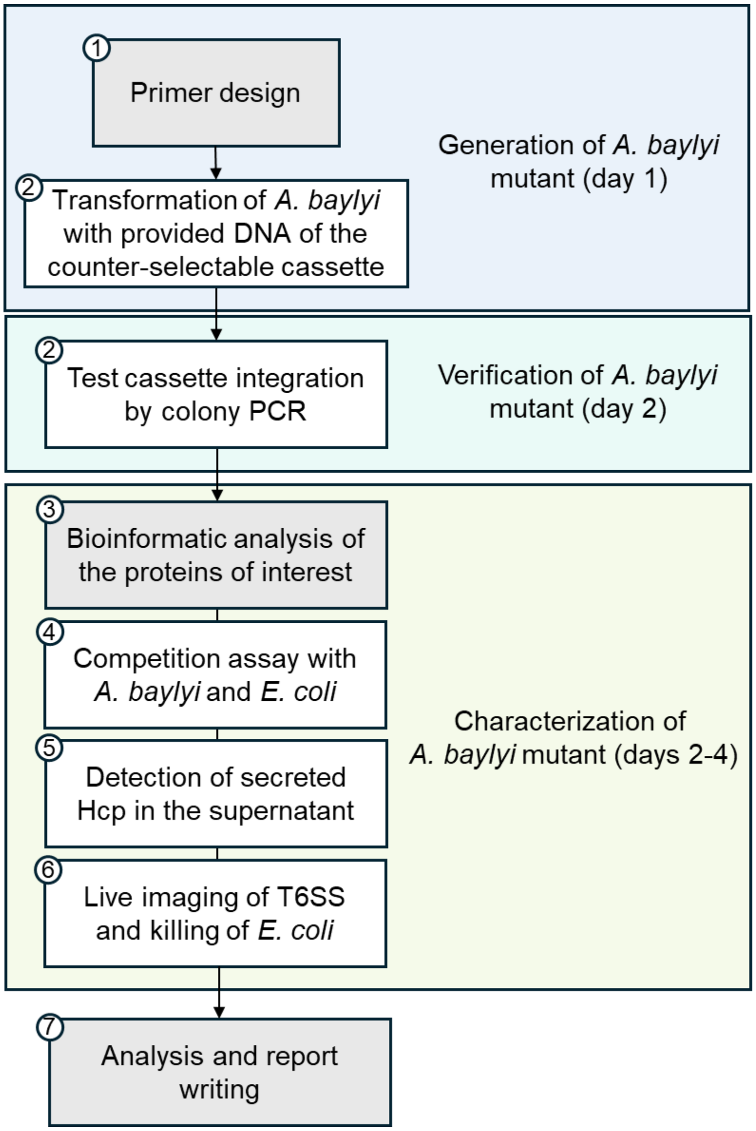

Figure 1: Workflow overview.

2 Lecture 1: Introduction to the T6SS and *Acinetobacter baylyi*

2.1 Introduction to the T6SS

Bacteria use a wide range of secretion systems to transport cytosolic proteins out of the cell and, in some cases, even into other cells. The nine types of secretion systems serve many different functions[1]. For example, the Type II Secretion System (T2SS) secretes its cargo proteins to the extracellular space, and the Type III Secretion System (T3SS) can establish a channel between bacteria and adjacent eukaryotic cells through which the bacterial cell is able to pump effectors into the cytosol of the eukaryotic cell. Secretion systems often play important roles in the pathogenesis of bacteria[2].

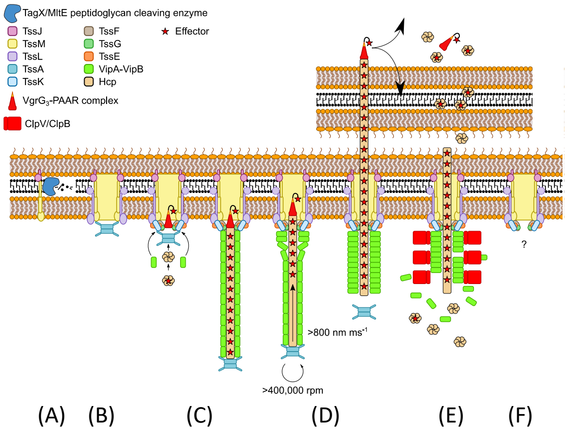

Figure 2. Model for T6SS assembly, effector translocation and component recycling. (A) A membrane complex (TssJLM) initiates formation of a baseplate (TssKEFG) with (B) spike proteins (VgrG/PAAR) and effectors. (C) This initiates polymerization of Hcp inner tube and VipA/VipB outer sheath. (D) Contraction of the sheath pushes the Hcp-tube with the spike and effectors into target cells and (E) the sheath is disassembled by ClpV. (F) The baseplate complex is ready for recycling or disassembly[10].

T6SSs are composed of a core of 13 conserved proteins and several associated proteins that differ between species. The T6SS is composed of three major structures: (i) membrane complex, (ii) baseplate and (iii) sheath-tube polymer (Figure 2). Of the characterized secretion systems, the T6SS is by far the largest, with a cytoplasmic sheath-tube that can span a whole cell and reach a length of up to several micrometers[3].

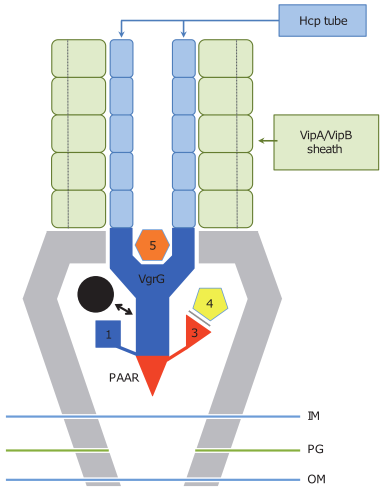

Figure 3. Proposed mechanism of effector binding and delivery (MERV model) [3].

The sheath-tube is composed of 3 proteins, of which two make up the sheath, VipA and VipB, and one forms the inner tube, Hcp. The tip of the Hcp-tube is composed of VgrGs and PAAR proteins (Figure 3). Both VgrGs and PAAR proteins are essential for assembly of the machinery and, in some cases, contain additional effector domains or bind cognate effectors[4]. VgrGs, PAAR proteins and effectors form a spike surrounded by a baseplate complex, which serves as a platform for initiation of the polymerization of the VipA/VipB sheath around the inner Hcp-tube. The assembly of a micrometer-long tail takes about 30 s. The sheath was shown to contract in less than 2 ms upon a trigger from the baseplate and by doing this, pushes the inner Hcp-tube with the cargo (VgrG, PAAR and associated effectors) out of the cell (Figure 2 and Figure 3)[5]. The effectors are injected into the prey cell, and the contracted sheath is specifically disassembled in approximately 30 s by an ATPase called ClpV, which binds to the N-terminus of VipB. Afterwards the baseplate can either be disassembled or recycled for the next round of assembly.

2.2 Introduction to *Acinetobacter baylyi*

Acinetobacter are Gram-negative, non-flagellated, and strictly aerobic environmental γ-proteobacteria. *Acinetobacter* strains are abundant in terrestrial habitats and are likely to make a significant contribution to the turnover of phytochemicals in the environment[6]. *Acinetobacter* are known for their metabolic versatility, as well as their contribution to the biodegradation of different pollutants and the extra- and intracellular production of a number of economically valuable products. *A. baumannii* strains have a narrower nutritional spectrum and are an increasingly important health problem, because they are often resistant to many different antibiotics and can cause nosocomial infections that are hard to treat. *A. baylyi* is a soil bacterium used to study metabolism and competence. The genome of *A. baylyi* is comprised of 3.6 Mbp, encodes 3325 genes, and its average GC content is 40%. Even though *A. baylyi* lacks genes known to be involved in pathogenesis, such as toxins and invasins, it contains several hemolysin-like proteins[7]. In Switzerland, *A. baylyi* is classified as a BSL1 organism.

A. baylyi is highly competent, allowing easy genetic manipulation[7]. Genes involved in natural competence are located on several clusters (*comFECB*, *comQLONM*, *pilBCD*, and *pilUT*). Transformation can be achieved by simple mixing of a log-phase *A. baylyi* culture with DNA (either a plasmid, a chromosomal DNA fragment, or a PCR product). It is possible to relatively easily and quickly construct a broad variety of chromosomal mutants, including marked and unmarked insertions, deletions, and replacements. *A. baylyi* can express a variety of foreign genes including antibiotic resistance cassettes, essential metabolic genes, negatively selectable catabolic genes and even intact operons from highly divergent bacteria[8].

Importantly, *A. baylyi* contains one highly active T6SS cluster (Figure 4), which it can use to kill competitors such as *E. coli*[9].

The *A. baylyi* T6SS cluster ($T6SS^{ADP1}$) contains all core genes that are present in all functional T6SS clusters such as Hcp, VgrG, PAAR, VipA, VipB, ClpV, TssEFG, TssKLM[10]. Additionally, $T6SS^{ADP1}$ contains an accessory protein TagF, which was found to be a posttranslational repressor of the H1-T6SS in *P. aeruginosa*[11]. Interestingly, proteins of unknown function ACIAD2693 and ACIAD2698 are exclusively found in the *Acinetobacter* genus. Potential effectors (Figure 4, in orange) were identified downstream of *vgrG* or PAAR genes and their functions were predicted by various programs such as HHPred[12]. Several adjacent genes were predicted to be potential immunity proteins (Figure 4, in purple).

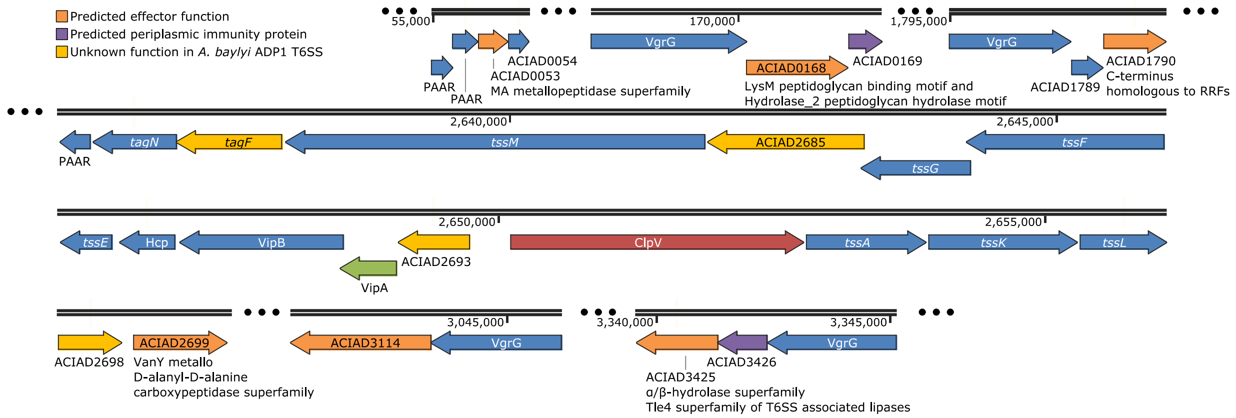

Figure 4. T6SS-related gene clusters in *A. baylyi* ADP1.

We have previously generated an *A. baylyi* strain in which VipA is fused to superfolder green fluorescent protein (sfGFP) and ClpV is fused to mCherry2[13]. Imaging of this strain with fluorescence microscopy reveals that the T6SS is highly dynamic (Figure 5).

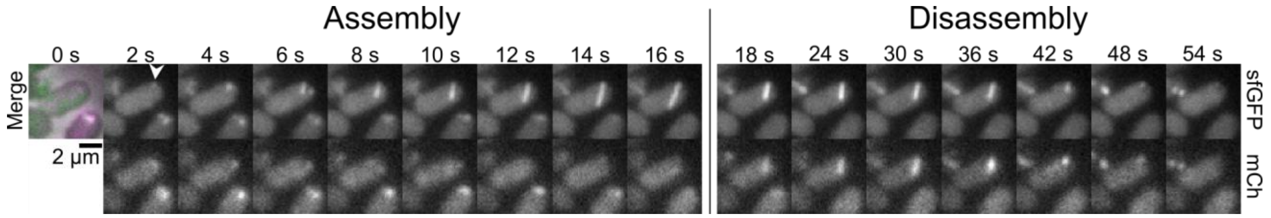

Figure 5. Dynamics of the T6SS in *A. baylyi* ADP1. Timelapse snapshots showing the assembly (left) and disassembly (right) processes of VipA-sfGFP. The disassembly also observed through the punctate localization of the ClpV-mCh2 fusion (right, lower panel).

3 Lecture 2: Mutagenesis in *A. baylyi*

3.1. Principles of mutagenesis in *A. baylyi*

To test the roles of various genes in T6SS dynamics and function, a modification of a previously published method for the generation of markerless in-frame deletions in *A. baylyi* will be used[8] (Figure 6).

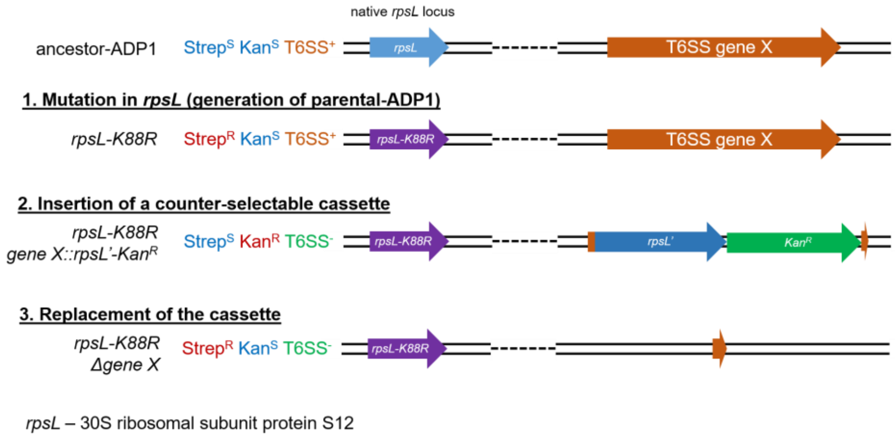

Figure 6. Principle of selection and counter-selection in *A. baylyi* ADP1.

To allow for selection of desired mutations, a streptomycin resistant *rpsL* mutation (K88R) was first introduced into the chromosome of *A. baylyi*, rendering it resistant to streptomycin (Strep^R). This bactericidal aminoglycoside antibiotic interferes with the accuracy and fidelity of the ribosome[14]. However, in cells harboring *rpsL*-K88R, the fidelity of the ribosome is close to wild type even in presence of streptomycin at concentrations up to ≈200 µg/mL. Various spontaneous mutations of the *rpsL* gene can be selected for in a population by growing *A. baylyi* in the presence of streptomycin.

Furthermore, we generated a genetic cassette containing an aminoglycoside phosphotransferase gene conferring kanamycin resistance (Kan^R) and a gene encoding the wild-type RpsL (streptomycin sensitive, *rpsL*'). Insertion of this *rpsL'*-Kan^R cassette to any location on the chromosome renders the *A. baylyi* strain kanamycin resistant but also streptomycin sensitive, because **reintroducing the wild-type RpsL dominantly confers streptomycin sensitivity**. Wild-type RpsL incorporates into ribosomes and, in presence of streptomycin, translation is obstructed, which leads to cell death. This allows for selection of replacement of the *rpsL'*-Kan^R cassette with a desired sequence, because strains that contain only the chromosomal *rpsL*-K88R allele are streptomycin resistant. This *rpsL'*-Kan^R cassette is therefore counter-selectable (selection for the loss of the cassette).

For efficient insertion of a DNA fragment into the chromosome of *A. baylyi* by homologous recombination, about 500 to 1000 bp of homology are required on both the 5’ and the 3’ end of the DNA fragment to be inserted. Desired DNA fragments containing the *rpsL'*-Kan^R cassette and homologous sequences are generated by overlap extension PCR (Figure 7 and Figure 8).

3.2. Principle of overlap extension PCR (OE-PCR)

As in most PCR reactions, two primers (one for each end) are used per sequence. To join two DNA molecules, special primers are used at the ends that are to be joined. For each molecule, the primer at the end to be joined is constructed such that it has a 5' overhang complementary to the end of the other molecule. Following annealing, when amplification occurs the DNA is extended by a new sequence that is complementary to the molecule it is to be joined to. Once both DNA molecules are extended in such a manner, they are mixed and a PCR is carried out with only the primers for the far ends. The introduced overlapping complementary sequences will serve as primers and the two sequences will be fused (Figure 7).

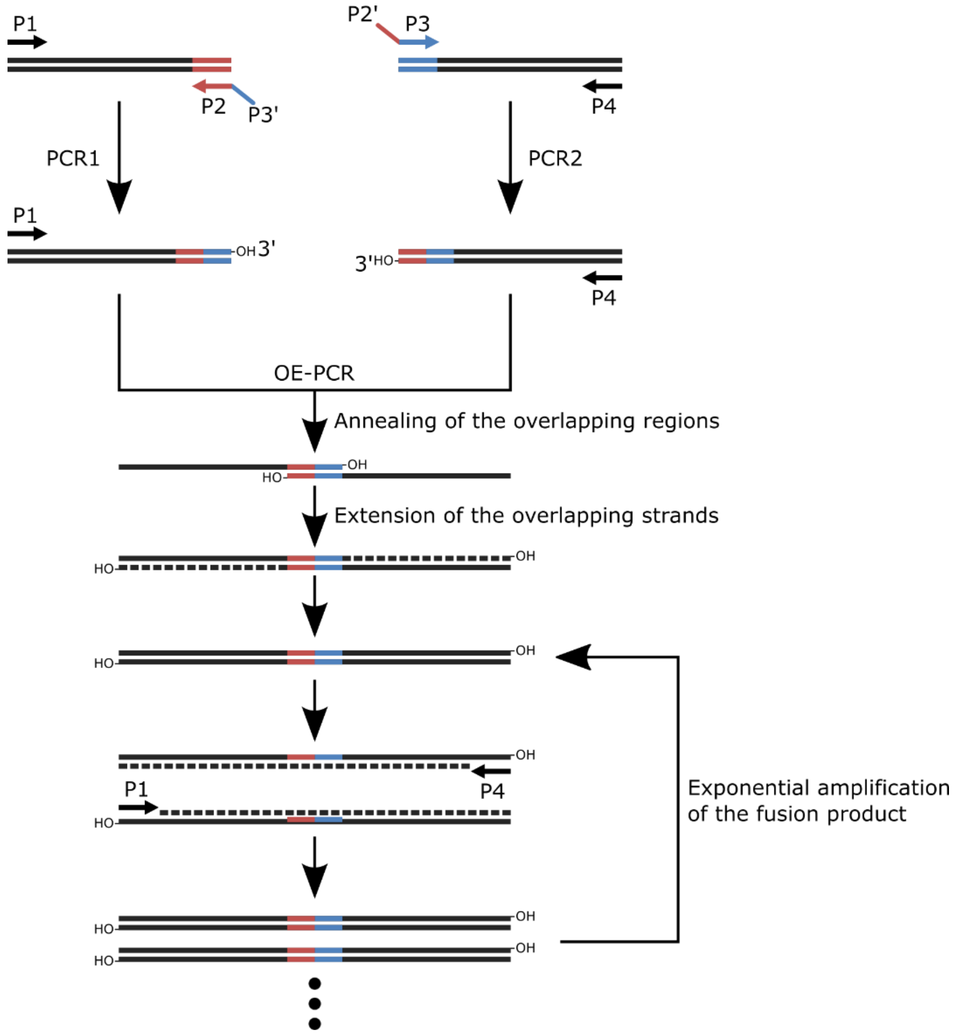

Figure 7. Scheme of Overlap Extension PCR.

4. Experiment 1: Primer design

4.1. Theory

Short DNA oligonucleotides (primers) are needed by DNA polymerases to initiate DNA synthesis. By defining the sequence of a primer, the sequence to which the primer anneals can be chosen and thus also the region that will be amplified by a DNA polymerase in a polymerase chain reaction (PCR). Because only 15-25 bases of the 3'-end of a primer have to be fully complementary to the target sequence for annealing, extra bases on the 5' end of the primer can be added to add new features (e.g. peptide tags for protein purification, DNA restriction enzyme recognition sites, protease cleavage sites) or to allow annealing with another DNA fragment.

Here, your goal is to design primers, according to the scheme in Figure 8, to synthesize DNA fragments for mutagenesis of *A. baylyi*. In general, your primers should have at least 20 bases of homology to the target sequence, a GC content of 40-60%, and a calculated melting temperature of 45-65 °C. Further, it is better if your primer has a couple of G or C bases at the 3'-end. You will design primers **P1-P6, D1 and D2, as well as S1 and S2 (see Figure 8)**. The primers should be specific to your target gene. Primers will be designed using SnapGene Viewer software (from GSL Biotech; freely available at www.snapgene.com).

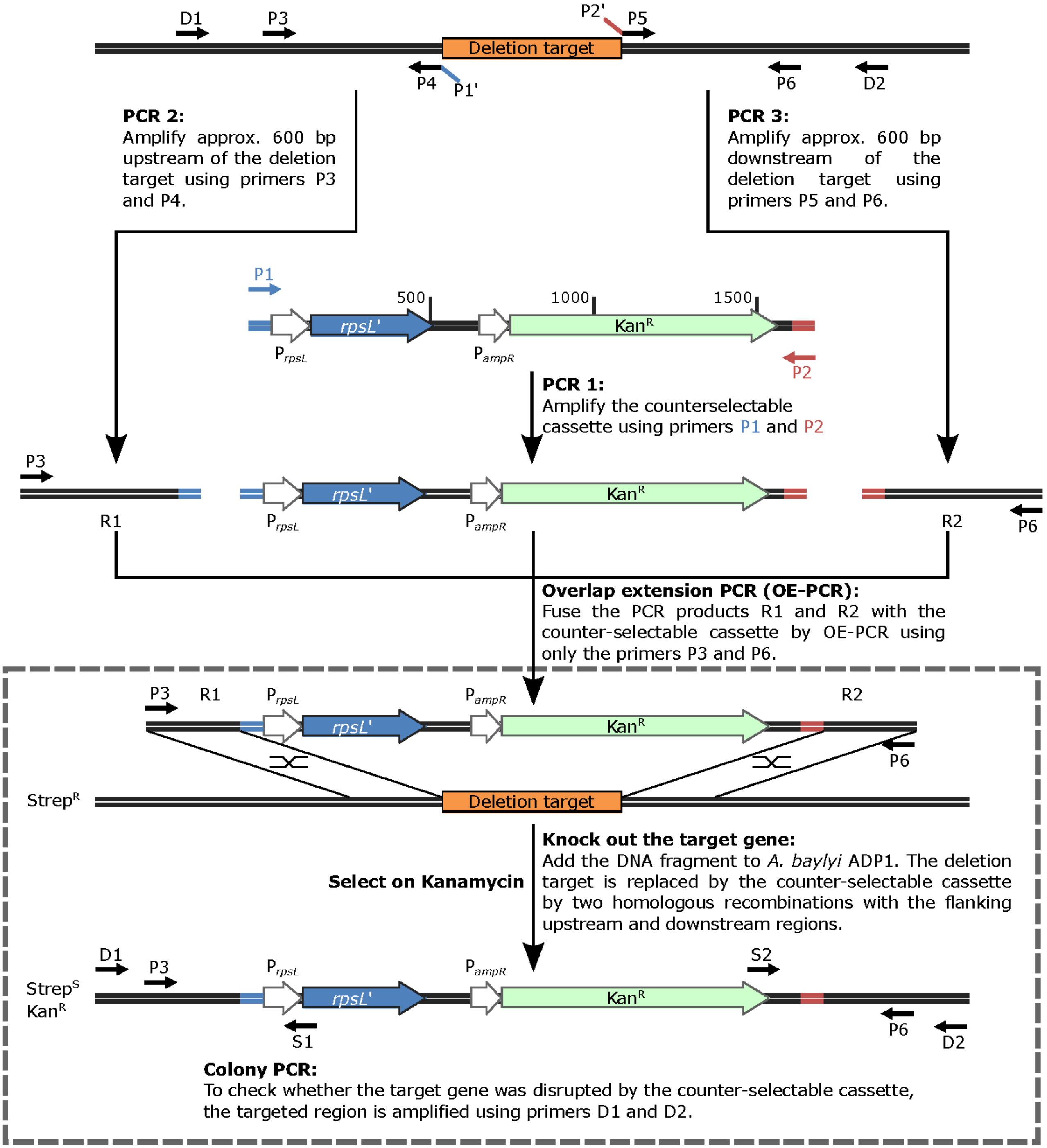

Figure 8. Cloning scheme for chromosomal knockouts in *A. baylyi*.

P1 – complementary to the 5’-end of the *rpsL’*-Kan^R cassette (For).

P2 – complementary to the 3’-end of the *rpsL’*-Kan^R cassette (Rev).

P3 – 500 – 700 bp upstream of the deletion target (For).

P4 – complementary to 5’-end of the deletion target (Rev). Additionally, the 5’-end of the primer contains 20-25 bases long sequence that is complementary to the 5’-end of the *rpsL’*-Kan^R cassette.

P5 – complementary to 3’-end of the deletion target (For). Additionally, the 5’-end of the primer contains 20-25 bases long sequence that is complementary to the 3’-end of the *rpsL’*-Kan^R cassette.

P6 – 500 – 700 bp downstream of the deletion target (Rev).

D1 – about 80-150 bp upstream of P3 primer (For). Used for sequencing and detection of desired mutation.

D2 – about 80-150 bp downstream of P6 primer (Rev). Used for sequencing and detection of desired mutation.

NOTE: Because of time limitations, in the first experimental step (section 6.1), we provided you with the construct containing the *rpsL’*-Kan^R cassette with flanking regions from genomic DNA of a previously generated strain using functional primers P3 and P6.

After transformation, we will verify the correct insertion of the *rpsL’*-Kan^R cassette by colony PCR in your generated mutant. Functional primers will be provided.

4.2. Primer design protocol

Gene to knock out	Function	Students
**ACIAD2685**	unknown	1-4
**ACIAD2689**	Hcp	5-8
**ACIAD2693**	TslA	9-12
**ACIAD2699**	TagX	13-16
**ACIAD3425**	putative lipase	17-22

Open the appropriate .dna file (ADP1_T6SS_main_cluster.dna for students 1-16 and ADP1_ACIAD3425_cluster.dna for students 17-22; RpsL-KanR.dna for the cassette for all) in SnapGene Viewer.

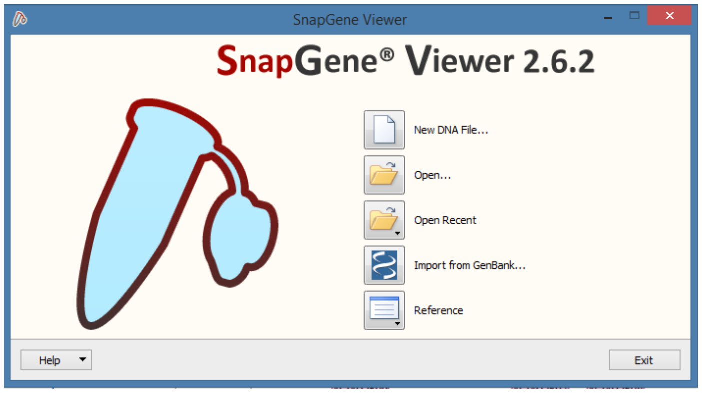

Navigate to “Sequence” tab (in the bottom of the window).

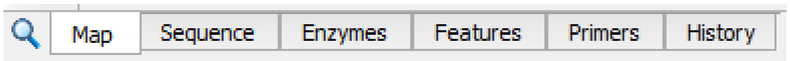

Select desired sequence by mouse to display the sequence length and approximate Tm. GC content is shown above the main window.

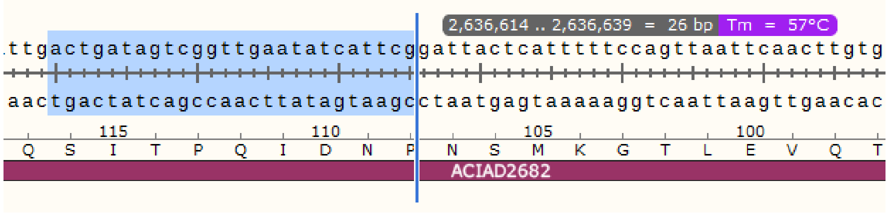

Select menu “Primers” – “Add Primer”, or press CTRL+R.

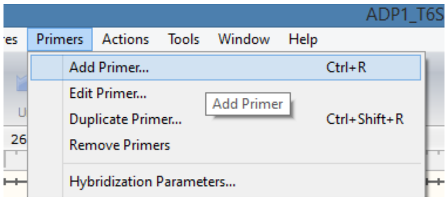

Select from which strand the primer should be generated.

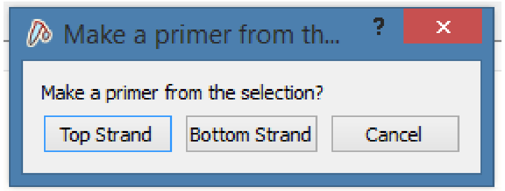

Name the primer according the scheme provided in Figure 8 and include the gene name.

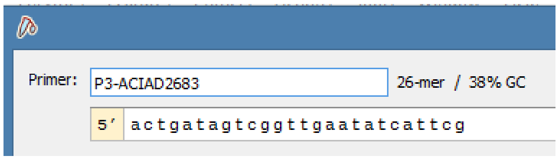

Click “Add primer to Template”.

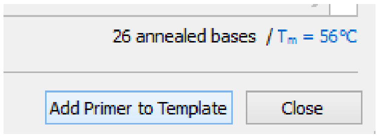

Primer will be shown on the sequence.

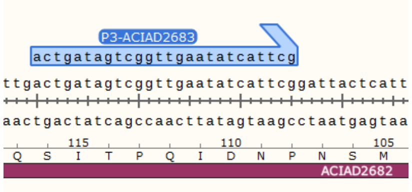

You can always edit the primer later by double-clicking on the primer. This will show you all the information about the primer (<u>NOTE: you should always check the length, Tm, and GC content here</u>).

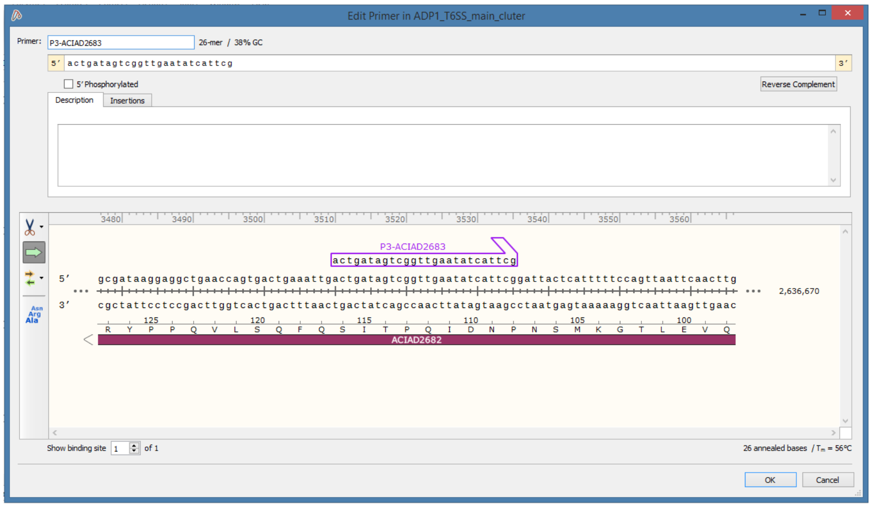

Continue designing additional primers.

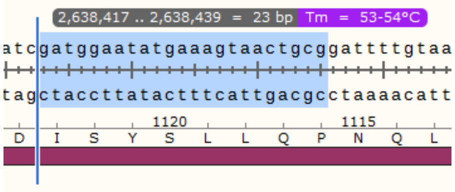

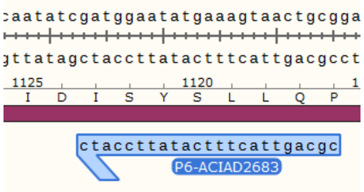

If you have designed two primers that are compatible for PCR (correct orientation), you can display the PCR product (and length) by selecting the primers in the “Map” tab while holding the Shift key.

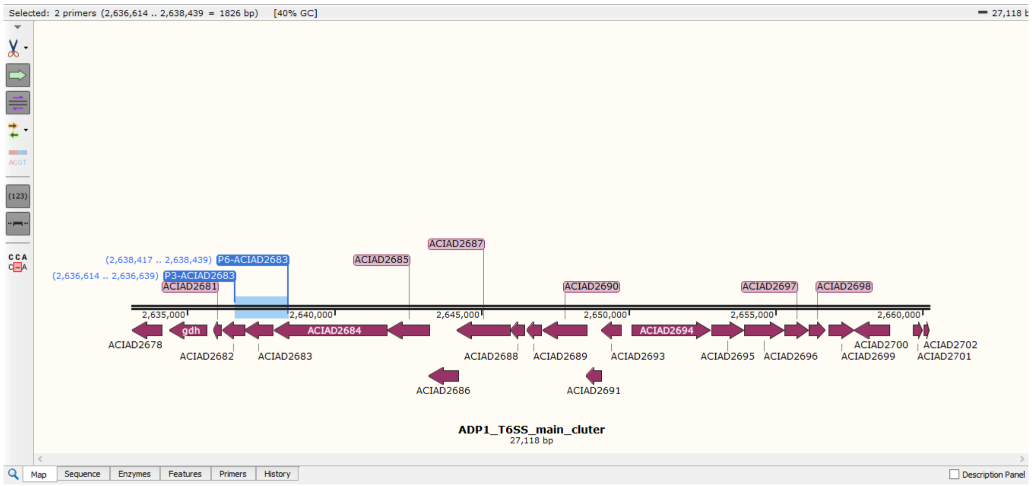

To add a DNA sequence that will allow to join PCR fragments by overlap extension PCR, double click on the primer to edit its sequence.

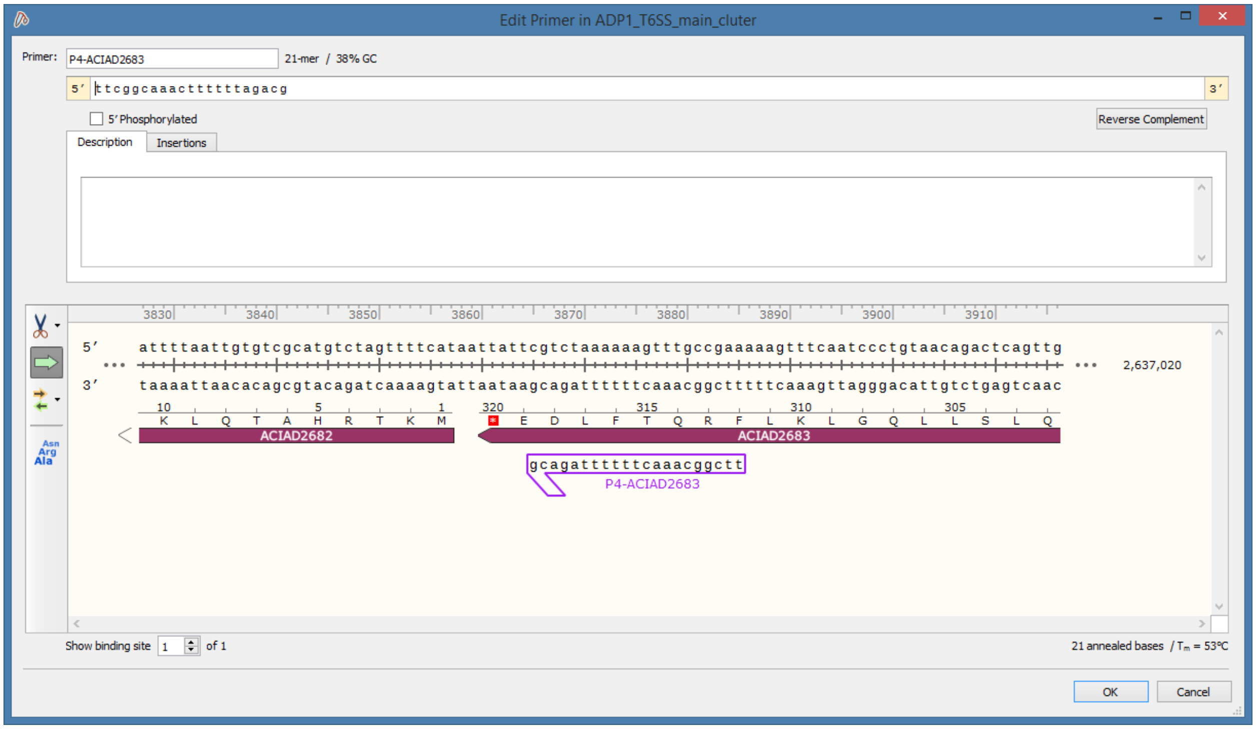

Paste the appropriate sequence to the 5’-end of the primer.

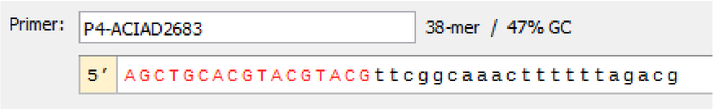

Notice that GC content and length as well as primer depiction is updated automatically.

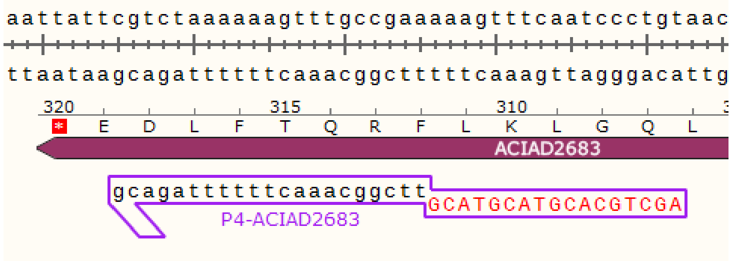

In the “Map” tab, select the region that will be amplified according to scheme in Figure 8.

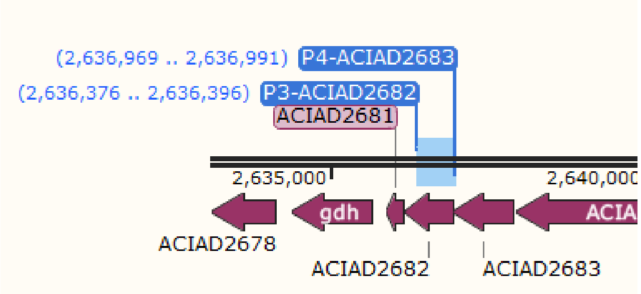

Click on “Features” – “Add Feature” or press CTRL+T.

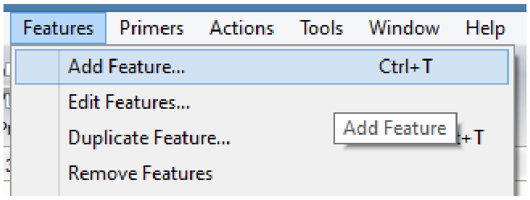

Rename the feature according to the scheme. You can change the color as well.

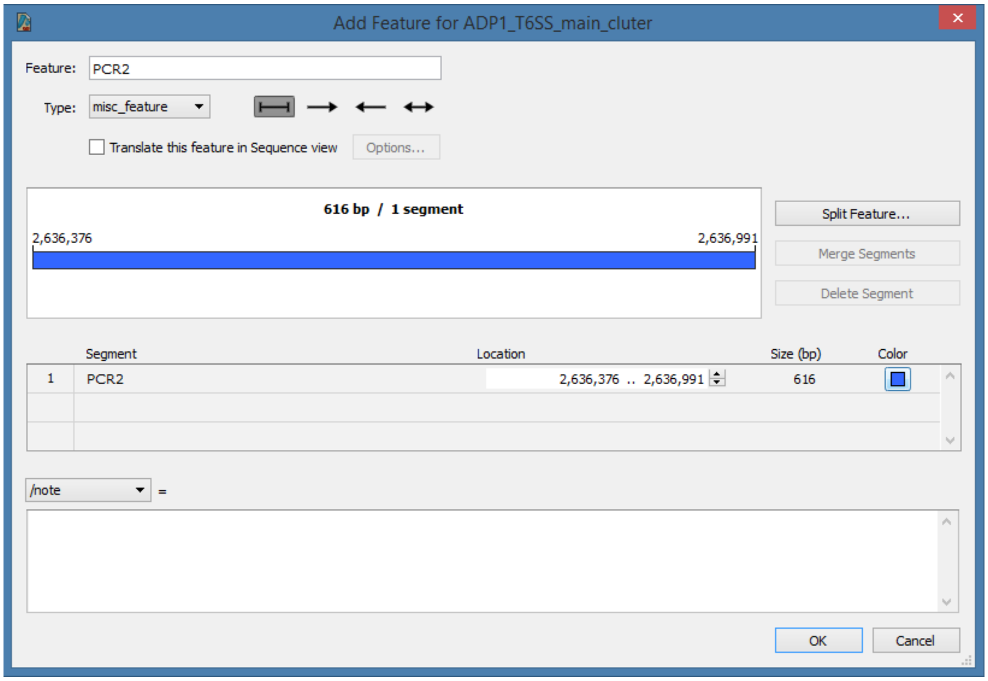

Add all 4 PCR fragments according the scheme in Figure 8. Save the SnapGene files as you will need them for making the figures for the report!

5. Experiment 2: Protein bioinformatics

Introduction

In this assignment, we will continue working with sequences, but instead of looking at DNA, we will move towards the products of the genes we have been discussing and work with the proteins themselves. In molecular biology, many research questions hinge on understanding the function of proteins, whether by themselves or in combination with other proteins or small molecules. Several decades of continuous research of proteins and computational methods have resulted in the existence of large databases and analytical tools which may help us design the experiments to understand our proteins.

Example

You have a car and you need to find out what it is best used for. Obviously, you could race it, take it off-road, try putting an entire apartment of Ikea furniture in it, etc... But ideally you would reduce the number of experiments you need. You know the name of the engine, the body shape and the ID number of the tires. In this case you would use existing databases of cars previously produced and perhaps find that the engine number and tire ID are similar to ones that are often seen in racing cars. Moreover, you know that the car is a coupe. At this point, it is likely to be a racing car. Obviously, you still need to take it on the track and figure out if that is correct, but the databases allowed you to narrow your search to a specific range of experiments.

Objective

You will attempt to generate as much predictive data as you can about the proteins encoded by the genes you are working with. Below you will find a list of tools that will help you put together as much detail about the functional/structural role of your proteins to help with future experiments. The main pieces of information you have are your amino acid sequence and the system you believe this protein is involved in (Type VI secretion systems).

Note: Using these tools generates predictions, not results! Even the most reliable predictions can be wrong when facing the harsh reality of life.

Tools

Expasy-translate: https://web.expasy.org/translate/

This is a tool that will translate a nucleotide sequence to an amino acid sequence. Please note that some of the translation options will indicate methionines as "Met" and if you copy paste a sequence with that definition it will mess up your other work.

Protparam: https://web.expasy.org/protparam/

This is a tool that provides basic information about an amino acid sequence such as molecular weight, extinction coefficient and the amino acid composition.

BLAST: https://www.uniprot.org/blast/

This is a tool that will align your sequence with other sequences in a large database and provide you with the closest hits. Depending on if your protein is already in a database, you will find your exact protein or very closely related homologs (<10% difference). When working on a new protein, database searches like this help identify potential roles of the target protein by comparing it to the function of identified homologs (usually <30% difference).

AlphaFold: https://alphafold.ebi.ac.uk/

A recent revolution in protein structure prediction, AlphaFold, developed by DeepMind and EMBL-EBI is a new deep-learning based structure prediction model that was able to significantly improve *in silico* protein models. In previous years Block Course students would use the Swiss-Model server (https://swissmodel.expasy.org/) to generate a structure prediction of their protein based on close homolog/homologs in the protein database (>30% similarity). However, since AlphaFold can take a long time to complete a prediction, we are instead providing you with a repository of completed predictions which you will use for your work.

NCBI Conserved Domain Search: https://www.ncbi.nlm.nih.gov/Structure/cdd/wrpsb.cgi

Since a lot can be learned about a protein from similar proteins, tools like this one are used to find conserved protein domains which might give insight into the function of your target protein.

TopCons: http://topcons.cbr.su.se/

As you already know, some proteins work while being embedded within membranes. This software will predict if your protein is one of them.

SignalP-6.0: https://services.healthtech.dtu.dk/services/SignalP-6.0/

Proteins that function in specific compartments/locations (such as the periplasm) need to be directed to these areas. Often an amino acid sequence will contain a leader peptide (short string of amino acids) that acts as “luggage tag” to tell the system where the protein belongs. This software will predict if your protein has one of these tags. (Don’t forget to select what kind of organism you have, Gram-positive, Gram-negative, eukaryote, etc.)

<u>Assignment</u>

Please use the tools above to obtain the following information about your gene of interest. Follow the instructions in the “report preparation” section to know what information to use in your report.

1. Copy the nucleotide sequence of your gene of interest from Snapgene file you work on for primer design.

2. Use **Expasy-translate** to generate translated amino acid sequence (pay attention to the direction of DNA strand).
3. Use translated amino acid sequence to generate predictions using all other tools listed above.

Key notes:

- For each tool pay attention to the metrics which describe the quality of the prediction. With that in mind, write a few sentences about what you learn from each tool and how useful it is in your case.
- For AlphaFold, look up the structure prediction of your gene using the gene ID, take an image of the structure for the report and note how much of the prediction seems to be reasonable or not.

General notes

The analytical software tools we have put here should function properly and be fairly straightforward to use, however if a server is down or a website is not responding please let us know and we will help. Some of them, like the BLAST suite, have a lot of options but you do not have to worry about them. In nearly all cases, simply putting your sequence and hitting "run" will be enough! The output pages can also be quite overwhelming but do your best to use the "help" options in the software suites themselves to make your conclusions.

6. Experiment 3: Chromosomal knockout in *A. baylyi*

6.1. Transformation of *A. baylyi* ADP1 with counter-selectable cassette by PCR

A. baylyi is a naturally competent strain, as it is able to take up extracellular DNA utilizing its competence machinery and incorporate it into its chromosome by homologous recombination. A $T6SS^+$, streptomycin resistant *A. baylyi* encoding VipA-sfGFP and ClpV-mCherry2 will be used as a parental strain. Here, for the sake of time, we provide you with the PCR-amplified DNA fragments (using P3 and P6) containing the *rpsL'*-Kan^R counter-selectable cassette with the homologous flanking regions that are necessary for the subsequent homologous recombination. You will transform this DNA fragment in the provided *A. baylyi* strain. The resulting mutants will be selected by plating on kanamycin plates.

6.1.1. Materials

- Sterile glass tubes

6.1.2. Reagents

- LA/Kan50-plates
- LB
- Culture of *A. baylyi* parental strain (provided by supervisors)
- DNA fragments to be transformed (provided by supervisors)

6.1.3. Method

1. An *A. baylyi* parental strain culture of the right OD_{600} will be provided by the supervisors in a glass tube. Label it with your name or group number. Write down the OD_{600} value (provided).
2. Add 10 µl of the provided counter-selectable cassette PCR product to the cultures and incubate them at 37 °C and 200 rpm for approx. 2 h.
3. Plate out 50 µl of the culture on an LB/Kan50 plate.
4. Transfer the rest of the same culture to a sterile 1.5 ml Eppendorf tube and centrifuge it for 1 min at full speed.
5. Discard the supernatant and resuspend the pellet in 100 µl LB.
6. Plate the cell suspension on the prepared LA/Kan50 plate and incubate at 37 °C overnight.
7. Next day: check the transformation plates and calculate the efficiency of recombination in *A. baylyi* in Colony Forming Units per ml (CFU/ml). Each student should count ¼ of a plate containing well spread single colonies if there are many colonies. An exponentially growing culture at an OD_{600} of 1.0 contains around $8x10^8$ CFUs/ml.

6.2. Colony PCR

To test if a correct mutant *A. baylyi* strain was generated, you will perform colony PCR on Wednesday. You will directly use the cells from the colony as a template for the PCR reaction. This is possible because the cells lyse during the initial denaturation step of the PCR and release their genomic DNA (gDNA). In addition, you should add an appropriate control to your experiment.

Your goal is to come up with a protocol for this experiment. Think about what you have learned during the lecture on principles of mutagenesis in *A. baylyi*, primer design and PCR. This should help you to select the correct cycling conditions for your PCR reaction. The Q5 DNA polymerase will be used and a section of the product sheet can be found below. This should help you to prepare the correct PCR master mix. A few practical tips for how to prepare the PCR reaction are provided bellow. Before you start, answer the following questions:

- Which primers do you choose for the detection of the insertion of your *rpsL'*-Kan^R cassette?
- What is a feasible annealing temperature? Choose two temperatures.
- What is the theoretical length of the PCR products amplified from wild-type and mutagenized strains? What is the optimal elongation time?
- Calculate the necessary volumes of each reagent for your PCR for 20 µl aliquots.
- What is your mutant control for PCR? What is your wild-type control?

Discuss and show your experimental design to your assigned supervisor before starting the experiment. You will then be provided with the necessary materials to perform this experiment.

COMPONENT	25 µl REACTION	50 µl REACTION	FINAL CONCENTRATION
5X Q5 Reaction Buffer	5 µl	10 µl	1X
10 mM dNTPs	0.5 µl	1 µl	200 µM
10 µM Forward Primer	1.25 µl	2.5 µl	0.5 µM
10 µM Reverse Primer	1.25 µl	2.5 µl	0.5 µM
Template DNA	variable	variable	< 1,000 ng
Q5 High-Fidelity DNA Polymerase	0.25 µl	0.5 µl	0.02 U/µl
5X Q5 High GC Enhancer (optional)	(5 µl)	(10 µl)	(1X)
Nuclease-Free Water	to 25 µl	to 50 µl	

Note: Gently mix the reaction. Collect all liquid to the bottom of the tube by a quick spin if necessary. Overlay the sample with mineral oil if using a PCR machine without a heated lid

Amplification rate of Q5 DNA polymerase: 30 s/kb

Students 17-22 (ACIAD3425 lipase) will need different primers, since their PCR product has the exact same length as wild-type. Therefore, they will use a primer binding to the cassette and a detection primer at the corresponding locus in the genome. There won't be any PCR product in the control.

6.2.1. Materials

- Thermocycler
- Ice (for the enzyme)

6.2.2. Reagents

- ddH_2O
- 5x Q5 Buffer
- 10 mM dNTPs (thaw on ice)
- 10 µM detection Primers
- Q5-Polymerase (at the supervisor station)
- 1 µl of cell suspension of mutants
- 1 µl of cell suspension of WT ADP1 (WT control)
- 1 µl of cell suspension of insertion mutant ADP1 (mutant control with the same cassette)
- Forward and reverse primers

Primer	Sequence	T_M [°C]	Annealing temp. [°C]	PCR product WT/mutant [bp]
**ACIAD2685_Det6_For**	cgcgcagatttttggcaagt	60	61/±3°C	2943/3157
**ACIAD2685_Det6_Rev**	gcgatgagcggattgagatg	58		
**ACIAD2689_Det6_For**	tcgattcgggctgcaattaa	55	58/±3°C	1979/3271
**ACIAD2689_Det6_Rev**	agaggatgtgactggtcagga	59		
**ACIAD2693_Det6_For**	ggtcaatgcgacataacgtg	58	57/±3°C	3211/4222
**ACIAD2693_Det6_Rev**	ttgaccacctgcaccaatta	54		
**ACIAD2699_Det6_For**	tttctgcacctgccgaagaa	57	58/±3°C	2349/3234
**ACIAD2699_Det6_Rev**	acccatggtcaagtacagga	55		
**ACIAD3425_Det_For**	ggcctgagtgtcgtattgct	59	58/±3°C	-/1022
**PrpsL-R**	atggcaattctcccgttaat	55		

6.2.3. Method

1. Mark 3 colonies of your transformation plate, pick them with pipette tips and transfer them into individual Eppendorf tubes containing 50 µl LB. **Be careful not to poke the agar while picking colonies, since it will inhibit your PCR reaction!** Resuspend with the tips vigorously to detach the cells from the tip. You will use this cell suspension as a template for PCR and also to inoculate your overnight cultures and plates. Work as carefully as possible and avoid cross contamination.
2. Perform colony PCR on 3 colonies and prepare 1 mutant control reaction as well as 1 wild-type control (control strains grown on plates will be provided). Use two different annealing temperatures. Calculate the appropriate volume of each reagent and prepare a master mix, assuming 20 µl reactions. What is the correct order mixing the ingredients?

Reagent	Volume per reaction[µl]	Total reactions	Total Volume [µl]
**Total**	19		190

Calculate for one extra reaction to allow for pipetting error!

3. After agreement by your supervisor, prepare the mix on ice and adding the reagents in the correct order (you should have received all reagents in your -20ºC box, except the Q5 DNA polymerase, which you can obtain (when?) at the supervisor station)
4. Mix gently and then aliquot 19 µl in PCR tubes and add 1 µl of cell suspension and of your control as template. After preparing the PCR reactions, mix gently and briefly spin down. Label your PCR tubes on the side.
5. The supervisor will help you to set up the thermocycler and start the PCR. The annealing temperature is chosen based on the Tm of the primers, the polymerase and the buffer. NOTE: During the initialization step, the cells lyse and release DNA. It is therefore better to prolong this step to 10 minutes to guarantee complete cell lysis.

Step	Temperature [°C]	Time [sec]
**Initialization**	98	600

6.3. DNA gel

To verify the presence and size of the desired PCR product, an aliquot of the PCR reaction is visualized on an agarose gel.

6.3.1. Materials

- Transilluminator
- Gel electrophoresis apparatus
- Microwave oven
- Erlenmeyer flask
- Measuring cylinder
- Scale

6.3.2. Reagents

- TAE buffer
- Agarose
- RedSafe (DNA stain)
- 1 kb DNA ladder (NEB)
- 6x loading dye

6.3.3. Method

1. Preparing agarose gel (4 students sharing 1 gel (two combs) with 1 chamber; talk to tutors during the course before starting):
   - Dissolve 1.2 g agarose in 120 ml TAE buffer (= 1 % agarose gel) in an Erlenmeyer flask by heating in a microwave oven for 1.5 min (wear leather gloves and **watch out for boiling retardations**).
   - Cool the solution to approx. 60 °C under flowing water.
   - Add 6 µl of 20,000 x RedSafe (**obtained at gel station from tutors; wear gloves and do not contaminate your pipette!**) to the solution, swirl the flask gently (avoid bubble formation) to mix and pour the gel into the cast. Remove any bubbles using the comb or a pipette tip. Place two combs in the cast to create wells for loading samples (tutors will show you where to insert combs for one gel).
   - Wait for the gel to solidify.
   - Transfer the gel to the electrophoresis chamber, fill with TAE buffer until the gel is fully submerged and remove the comb.
2. Mix 5 µl of the PCR products with 1 µl of the 6x loading dye
3. Load the samples and 10 µl of the 1 kb DNA ladder on the gel and run it at 70 V for 1 h (4 students load own samples on one gel and run it together; talk to tutors during the course before it). (NOTE: under these conditions, DNA is negatively charged and will therefore migrate from the cathode to the anode. Make sure to connect the electrophoresis chamber to the power supply with the right polarity.)
4. Transfer the gel to the UV transilluminator and take an image of the gel (the image will have to be in your report).

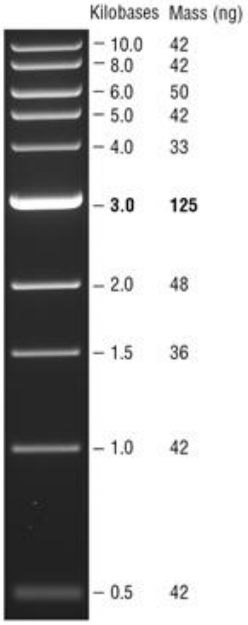

Figure 9. 1 kb DNA Ladder visualized on a 0.8% TAE agarose gel. Mass values are for 0.5 µg/lane.

Regrow one of the colonies that you are testing by PCR for further experiments:

6. After analyzing your colony PCR product on the DNA gel, regrow one of the tested colonies, verified by colony PCR (if possible), by plating 20 µL of the respective cell suspensions on individual LA-Kan50 plates. Incubate overnight at 37°C.
7. Use the remaining volume of the same cell suspensions from the same colony verified by colony PCR (if possible) to inoculate a 3 ml LB-Kan50 liquid culture. Incubate at 37°C and 200 rpm (kanamycin stocks will be provided by supervisors).

Your report should contain a brief explanation on the experimental procedure; include the annotated image of your DNA gel.

7. Experiment 4: Bacterial competition assay

7.1. Theory

The bacterial competition assay (killing assay) is a simple method to observe changes in the killing efficiency of different strains that attack another strain. In this block course, *A. baylyi* – with different chromosomal mutations – serve as attacking strains and *E. coli* MG1655 serves as a prey strain. The underlying principle of this method is that if *A. baylyi* kills *E. coli* very efficiently, the number of viable *E. coli* is largely reduced whereas in a competition with a killing-deficient *A. baylyi* strain, the number of viable *E. coli* should not be altered. Therefore, one can analyze how different mutations affect the function of the T6SS.

Several different methods exist to monitor the killing efficiency after co-incubation of the strains to be tested. **Qualitative methods** include plating the cultures on plates that contain a chromogenic substrate such as X-Gal (5-Bromo-4-chloro-3-indolyl β-D-galactopyranoside) or CPRG (Chlorophenol Red-β-D-galactopyranoside). Both of these substrates can be converted by β-galactosidase. X-Gal is converted into Galactose and 5-Brom-4-chlor-3-hydroxyindol, which in turn reacts with oxygen in the air to 5,5'-Dibromo-4,4'-dichloro-indigo, a blue chemical. CPRG is yellow and is converted to chlorophenol red, a red chemical. A major difference between the two substrates is their cell permeability; X-Gal is cell permeable whereas CPRG is not. This means that X-Gal can be converted by living cells and forms a blue precipitate, whereas CPRG can only be converted by β-galactosidase that leaked out of dying or dead cells and then forms a red halo around the dying cells. Chlorophenol red is water soluble and diffuses in the agar, and therefore needs to be visualized in time. <u>In our experiment, we use an *E. coli* strain expressing the *lacZ* gene that encodes β-galactosidase, whereas the *A. baylyi* strain lacks *lacZ*</u>.

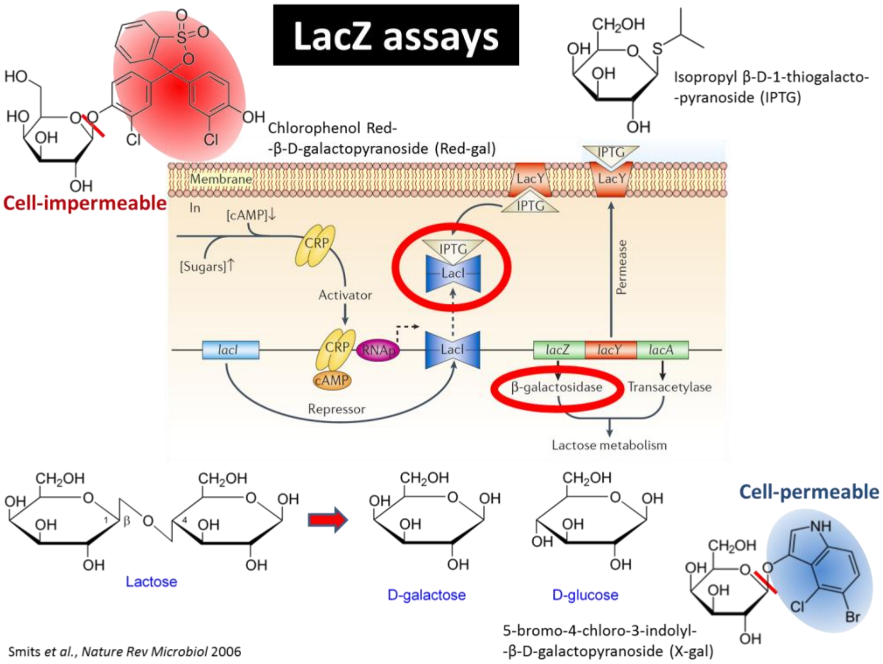

Figure 10. Overview of β-galactosidase detection assays.

Fill out the table to test if you understand the difference between the X-gal and Red-gal killing assay.

	X-gal (blue/yellow)	Red-gal (red/yellow)
E. coli		
ADP1		
**T6SS+** ADP1 + *E. coli*		
**T6SS-** ADP1 + *E. coli*		

To quantify the number of cells that survived during various interactions, one can do a more labor intense assay (**quantitative killing assay**). For this, after co-incubation of the strains under investigation, the bacterial mixture will be recovered from the plate and serially diluted. The dilutions will be plated on selective plates that only allow for growth of one strain to count CFU of surviving bacteria.

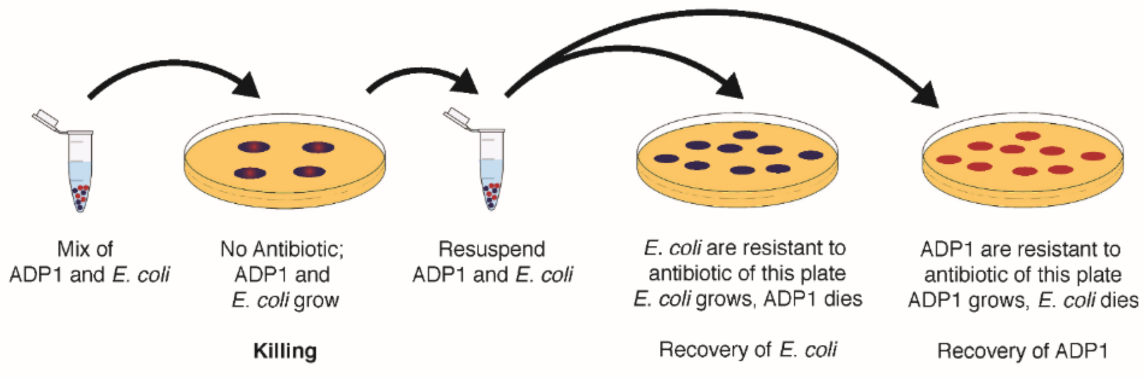

Figure 11: Overview of quantitative killing assay.

7.2. Protocol

7.2.1. Materials

- Cuvettes
- Spectrophotometer set to measure at 600 nm
- Sterile Pasteur pipettes
- Incubator

7.2.2. Reagents

- LA/X-Gal40/IPTG100 plates
- LB
- LA/CPRG20/IPTG100 plates
- LA/Gentamicin20 plates
- LA plates
- LA/Strep100 plates
- overnight cultures of mutant strains (streptomycin resistant) (provided by tutors)
  NOTE: the mutant strains used for competition assay and live-cell imaging are the in-frame deletion mutant of your genes of interest; for time limitation reasons, we are not able to continue generating in-frame deletion in this course as shown in Lecture 2; however, we will use such strains previously generated in the lab for the characterization of mutants.
- overnight cultures of *A. baylyi* parental strain (streptomycin resistant) (provided by tutors)
- overnight cultures of T6SS-deficient *A. baylyi* (streptomycin resistant) (provided by tutors)
- overnight cultures of *E. coli* MG1655 (gentamicin resistant) (provided by tutors)

7.2.3. Method

7.2.3.1. Killing assays

1. Transfer 1 ml of each overnight culture (*A. baylyi* mutants, *E. coli* prey, *A. baylyi* T6SS$^+$, and *A. baylyi* T6SS$^-$) to a sterile 1.5 ml tube.
2. Centrifuge all tubes for 2 min at maximum speed.
3. Discard the residual supernatants and resuspend each pellet in 1 ml LB.
4. Repeat the centrifugation step (2 min at maximum speed). (Think about why this washing step is important!)
5. Discard the supernatants and resuspend the pellets in 0.5 ml LB each.
6. In cuvettes, prepare 1 ml cell suspensions of your samples that are diluted 20-fold with LB and measure the OD_{600}.
7. From the measured OD_{600}, calculate the necessary dilution to obtain cell suspensions at an OD_{600} of 1.0. Then adjust the cell suspensions with LB accordingly.
8. Mix the following nine cell suspensions in new, sterile 1.5 ml tubes:
   a. 20 µl *A. baylyi* mutant with 20 µl *E. coli* prey (ratio 1:1)

b. 10 µl *A. baylyi* mutant with 100 µl *E. coli* prey (ratio 1:10)
c. 100 µl *A. baylyi* mutant with 10 µl *E. coli* prey (ratio 10:1)
d. 20 µl T6SS$^+$ *A. baylyi* with 20 µl *E. coli* prey (ratio 1:1)
e. 10 µl T6SS$^+$ *A. baylyi* with 100 µl *E. coli* prey (ratio 1:10)
f. 100 µl T6SS$^+$ *A. baylyi* with 10 µl *E. coli* prey (ratio 10:1)
g. 20 µl T6SS$^-$ *A. baylyi* with 20 µl *E. coli* prey (ratio 1:1)
h. 10 µl T6SS$^-$ *A. baylyi* with 100 µl *E. coli* prey (ratio 1:10)
i. 100 µl T6SS$^-$ *A. baylyi* with 10 µl *E. coli* prey (ratio 10:1)

9. For the **qualitative assays:** spot 5 µl of each of these mixtures as well as of the single strains (T6SS$^+$ *A. baylyi*, T6SS$^-$ *A. baylyi*, *A. baylyi* mutant and *E. coli*) in duplicate on a LA/X-Gal40/IPTG100 plate as well as on a LA/CPRG20/IPTG100 plate.
10. Allow the spots to dry and then incubate the CPRG and X-gal plates at 37 °C. Keep checking the plates approx. every 2h and take pictures if you see any color changes. Keep the plates overnight at 37 °C but make sure that you take at least one **picture of the plates the same day before you leave**. Annotated pictures should be in your report as the result of the qualitative assays!

11. For the **quantitative killing assays:** spot 5 µl of the 10:1 ratios (*A. baylyi* : *E. coli*) of the following combinations on a LA plate in duplicate. Avoid touching/poking the agar with the tip as *E. coli* prey will be protected from T6SS-dependent killing inside the agar.
    c. A. baylyi mutant vs. *E. coli* prey
    f. T6SS$^+$ *A. baylyi* vs. *E. coli* prey
    i. T6SS$^-$ *A. baylyi* vs. *E. coli* prey
12. Allow the spots to dry and incubate the plate at 37 °C for approx. 2-4 h.
13. Prepare six 1.5 ml tubes with 500 µl LB each.
14. Prepare 1.5 ml tubes (6 per spot) for serial dilution by adding 90 µl LB to each tube and label them accordingly.
15. Take the LA plate out of the incubator.
16. Carefully punch out both spots of each of the three different mixtures and transfer the agar discs to the previously prepared tubes with 500 µl LB. Use a new pipette tip for each spot. Your supervisor will show you how to perform this task.

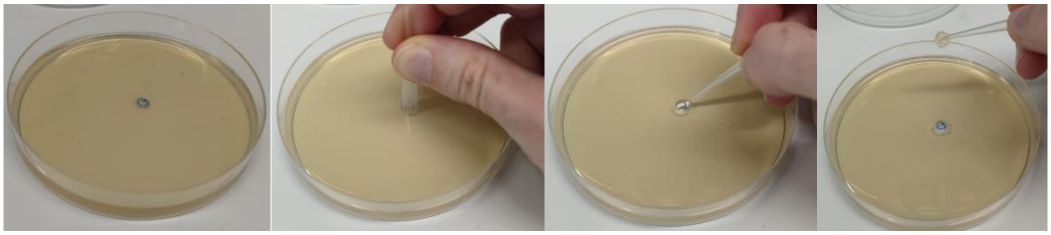

Figure 12: Steps of the spot retrieval process.

17. Resuspend the bacterial spots by vigorous vortexing (at least 30 s).
18. Transfer 10 µl of the suspensions to the corresponding tube for the first 10-fold dilution:
    Tube A: *A. baylyi* mutant strain vs. *E. coli* prey in a 10:1 ratio replicate 1
    Tube B: *A. baylyi* mutant strain vs. *E. coli* prey in a 10:1 ratio replicate 2
    Tube C: T6SS$^+$ *A. baylyi* vs. *E. coli* prey in a 10:1 ratio replicate 1
    Tube D: T6SS$^+$ *A. baylyi* vs. *E. coli* prey in a 10:1 ratio replicate 2
    Tube E: T6SS$^-$ *A. baylyi* vs. *E. coli* prey in a 10:1 ratio replicate 1
    Tube F: T6SS$^-$ *A. baylyi* vs. *E. coli* prey in a 10:1 ratio replicate 2
19. Perform serial 1:10 dilutions using the remaining tubes (ALWAYS use a fresh tip for each dilution step).
20. Spot 5 µl of each of these dilutions on a LA/Gent20 plate.
21. Spot 5 µl of each of these dilutions on a LA/Strep100 plate.
    Incubate these plates at 37 °C overnight.

The next day:

22. Take images of your killing assay plates (also X-gal and CPRG if necessary or if color changes are easier to see). Use these images in your report!
23. On the plates with dilution series, count the colonies of a dilution that contains countable number of colonies (approx. 5-30).
24. Calculate the CFUs for the surviving *E. coli* prey on LA/Gent20 plates. Calculate the CFUs for the *A. baylyi* strains on the LA/Strep100 plates (this is your control of proper pipetting and recovery of cells from the competition plate).

8. Experiment 5: Detection of Hcp secretion in the supernatant of *A. baylyi* ADP1 cultures

To identify proteins that are secreted into the medium by secretion systems, one can analyze the cell culture supernatants by SDS-PAGE (potentially followed by a Western blot) or by mass spectrometry. To reduce the sample volume and increase the concentration of the proteins to be analyzed, you will precipitate proteins. Precipitation is achieved by addition of trichloroacetic acid (TCA). TCA interacts with proteins, denatures the structured parts and keeps them in a molten-globule like state. Proteins in this state are significantly more prone to aggregation than properly folded ones. Precipitation seems to be independent of the physicochemical properties of the studied proteins and therefore has the same efficiency for all proteins[15]. The acetone in the protocol serves only for washing purposes.

Please refer to Chapter 10.2 for further information on the chemicals used in this experiment.

8.1. Materials

- Heat Block, preheated to 70 °C
- Table top centrifuge with cooling system (precool to 4 °C)
- Glass plates (large with spacers)
- Glass plates (small without spacers)
- Parafilm
- Combs
- Bio-Rad gel system
- Bio-Rad power supply
- MultiFlex fine tips for loading samples
- Graduate cylinders 100 ml, 500 ml and 1000 ml
- Boxes for staining and destaining
- Filter paper
- Shaker
- 50 ml tubes
- 15 ml tubes

8.2. Reagents

- LB
- Trichloroacetic acid (TCA) 100% (w/v)
- Acetone (precool on ice)
- Ice
- 30 % Acrylamide-Bis solution

Acetone:

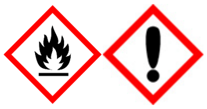

30 % Acrylamide-Bis solution:

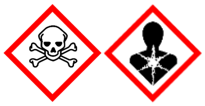

10 % APS:

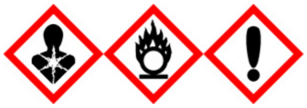

NuPAGE Antioxidant solution:

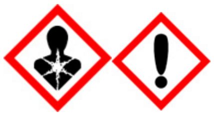

SeeBlue Plus 2:

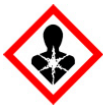

TCA:

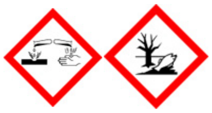

- 1.25 M Bis-Tris buffer pH 6.4 (3.5 x)
- 10 % APS solution
- TEMED
- LDS sample buffer (1 x)
- DTT (10x)
- MES running buffer (20 x)
- NuPAGE Antioxidant solution
- Protein size marker (SeeBlue Plus 2)
- Instant Blue gel staining solution

8.3. Method

8.3.1. SDS PAGE Gel

1. **Before you start a supervisor will demonstrate the assembly of the cassette once.**
2. Each group will run one gel and thereby prepare one together. The following list gives the composition for one gel.
3. Assemble a glass cassette using one large glass plate and one small glass plate. Use Parafilm to seal the bottom of the cassette. Mount the cassette into the Bio-Rad gel casting device and check for leakage by adding H_2O.
4. If the chamber seals well, drain H_2O by tilting the cassette over the sink. Dry residual H_2O with a filter paper.
5. In a separate 15 ml tube prepare the separating gel mixture (see table) by adding the reagents in the order shown in the table below.
   In case of spillage of the Acrylamide-Bis immediately contact a supervisor. Do not attempt to clean the spillage yourselves!
6. Immediately after the addition of 10% APS and TEMED vortex briefly to mix well and transfer the mixture into the cassettes with a P1000 pipette. The level of the gel mixture should be 8-10 mm below the comb (18-20 mm from the upper edge of the small glass plate; approx. 3.3 ml of gel mixture will be needed).
7. Gently add a layer of H_2O on top of the gel mixture to obtain a uniform horizontal interface (approx. 1 ml). Use a P200 pipette with a MultiFlex fine tip for this purpose. Let the gel polymerize for 30-60 min at room temperature.
8. When the gel has polymerized, remove the liquid from its upper part; use filter paper to carefully wipe residual buffer from the top of the gel. Do not touch the gel with filter paper.
9. Prepare the stacking gel mixture (see table below) and pour it on top of the separating gel. Add the comb carefully and let it polymerize (watch for spraying unpolymerized gel when inserting the comb **WEAR SAFETY GOGGLES**). Polymerization of the stacking gel

should be complete within 5-10 min. You should work **RAPIDLY** as this mixture will polymerize very soon after adding 10% APS and TEMED.

	Separating gel (10%)	Stacking gel (5%)
**H_2O**	2 ml	1.4 ml
**Bis-Tris Buffer 3.5x**	1.5 ml	0.75 ml
**30 % Acrylamide-Bis**	1.75 ml	0.45 ml
**10% APS**	20 µl	20 µl
**TEMED**	2 µl	2 µl

*IMPORTANT: only add 10% APS and TEMED right before you are ready to transfer the mixture to the cassette.

8.3.2. Hcp detection (four groups will share one tank to run the protein gels simultaneously.)

1. Set up a day culture for your ADP1 mutant strain, the ADP1 parental strain and the T6SS$^-$ control strain by diluting 1:25 into 3ml fresh LB without antibiotics at 37°C, 200 rpm for ~3.5-4h.
2. Proceed with killing assay 7 Experiment 4: Bacterial competition assay
3. From the day cultures, transfer 1 ml of your ADP1 mutant strain colony 1 and colony 2, the ADP1 parental strain and the T6SS$^-$ control strain each in a fresh 1.5 ml Eppendorf tube, centrifuge them for 1 min at maximum speed.
4. Transfer 900 µl of each culture supernatant in a fresh 1.5 ml Eppendorf tube.
5. For this precipitation step and all washing steps wear safety goggles and gloves as the used TCA is highly corrosive! Note: skin burns will leave a scar but eyes are irreversibly damaged. Add 100 µl 100% (w/v) trichloroacetic acid to 900 µl of the culture supernatant.
6. Incubate the samples for 10 min on ice, while mixing from time to time.
7. Centrifuge the tubes for 5 min at 14,000 rpm and 4 °C.
8. Transfer the supernatant to a waste tube (do not discard this supernatant into the sink! And never add to autoclaving waste (risk of thermal decarboxylation, corrosion of the autoclave and injuring the cleaning staff)) and keep the pellet intact. In the pellet, you will find the precipitated protein.
9. Wash the pellets with 500 µl ice cold acetone.
10. Centrifuge the tube for 5 min at 14,000 rpm and 4 °C.
11. Repeat steps 8-10 one more time.
12. Remove as much acetone as possible and let the pellets dry (with the lids open) for 10 min at RT.
13. Add 20 µl of 1x LDS (as provided) to each of the dry protein pellets from TCA precipitation and vortex well to completely dissolve the pellets, centrifuge at maximum speed for 1 min, then place samples on ice.
14. Add 2 µl of the 10x DTT solution to each sample, close tubes, vortex again, centrifuge briefly, then heat samples for 10 min at 70 °C in a heat block.

15. Prepare 1 liter of running buffer per running chamber by diluting the 20x MES solution to 1x MES buffer. Transfer 200 ml of this solution in a clean 200 ml bottle and add 500 µl of antioxidant (WARNING: the antioxidant is dissolved in DMF which is TOXIC).
16. Remove combs carefully and place gels into the Bio-Rad chamber (two per running chamber).
17. Filling with running buffer: 1x MES + antioxidant into the inner (cathodic) compartment and 1x MES into the outer (anodic) compartment.
18. Load the most left lane of the gel with 7 µl protein size marker SeeBlue Plus 2 and add your samples to the next free lanes. For loading the samples use MultiFlex fine tips. Please write down in which lanes your samples are.
19. Close the chamber lid and plug the chamber to the power supply (mind the correct electrical polarity!), and let it run at 150 V constant voltage until the blue front line reaches the bottom.
20. Unplug the chamber from the power supply, disassemble glass cassettes with the plastic wedge (provided) and gently transfer each gel into a plastic box containing 15 ml of Instant Blue gel staining solution (mix before pouring into box). ATTENTION: The polyacrylamide gel is very fragile and it may stick to one of the glass plates. To avoid breaking the gel during transfer it is advisable to submerge glass plate together with gel into H_2O and detach gel while submerged.
21. Shake gently at room temperature overnight.
22. On the next day, discard the staining solution and replace it with H_2O. Continue shaking gently at room temperature until the gel background has become clear and take a photo of the gel to obtain a permanent record of the result. Estimate molecular sizes of your protein bands by comparison with the bands of the Protein size marker.

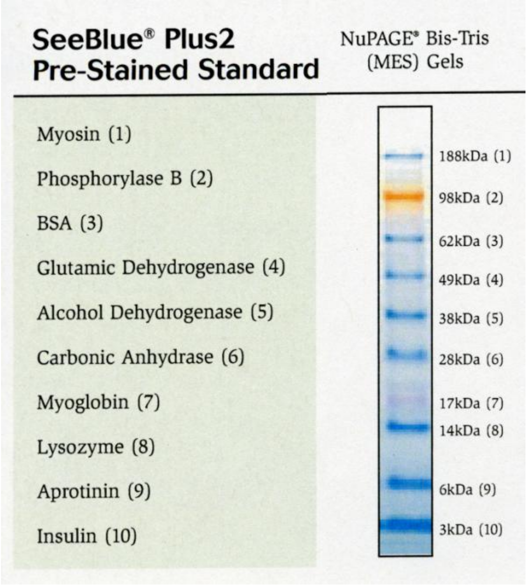

Figure 13. Apparent molecular weights of the SeeBlue® Plus2 Pre-Stained Standard.

9. Experiment 6: Imaging of T6SS in *A. baylyi*

To assess the ability of the mutated *A. baylyi* to assemble T6SS, you will image sfGFP-labeled VipA and mCherry2-labeled ClpV in *A. baylyi*. From the sfGFP and mCherry2 subcellular localization, you will be able to estimate the efficiency of T6SS assembly in your mutant, using your own high-resolution time-lapse microscopic videos. Furthermore, to dissect the contribution of individual mutations to the killing, you will observe the killing of *E. coli* cells, and you will analyze provided videos of how *A. baylyi* strains that contain only a single effector kill *E. coli* target cells. From morphological changes of the target cells you will hypothesize what the individual effectors do.

To image bacterial cells under a microscope, they need to be immobilized to avoid movement. To achieve this, you will prepare agarose pads on which the bacteria will stick. You will use a fluorescence microscope to observe your samples. Groups working on the same gene of interest will then collect images in a phase contrast transmission mode (to see the cells) and fluorescence mode for both parental control and your mutants. The images should be included in your report with a brief description.

You will also observe the killing of *E. coli* cells by mixing your mutant *A. baylyi* strain with *E. coli* and incubating them on an agarose pad that contains SYTOX blue, a DNA stain that is impermeable to the cell membrane and will therefore only stain those cells whose permeability is compromised, that is, cells that are dying and lysing. You will analyze provided microscopy videos of different *A. baylyi* mutants (Supplementary Videos 1-9) and hypothesize on their potential roles in the T6SS.

9.1. Materials

- Table top centrifuge
- Eppendorf tubes
- Pre-warmed heating plate to 95°C
- Microscope slides
- Cover slips
- Lens cleaning wipes
- Epifluorescence microscope
- Tweezers
- Timer

9.2. Reagents

- LB
- Immersion oil
- 1 % agarose in LB-PBS – melt the aliquots on time!!

- Propidium iodide 200 µg/mL
- Culture of *A. baylyi* parental strain
- Culture of *A. baylyi* mutant

9.3. Method

9.3.1. Killing of *E. coli* by *A. baylyi*

1. Your supervisor will show you how to prepare a thin agarose pad for imaging.
2. Now try it yourself, take a microscope slide and put 2 cover slides on the left and right side. In case it does not work properly, clean the slide and try again.
3. You will receive a reaction tube containing molten 1% agarose in LB-PBS and an aliquot of propidium iodide (dye that will stain dying cells).
4. Pipette 100 µl of the molten 1 % agarose in LB-PBS with propidium iodide in the middle of the microscope slide. Place the molten agarose tube back to the water bath; you will need it again later.
5. Put another microscope slide on top of the agarose and use the 2 cover slips on each side as spacers. This has to be done **FAST** before the agarose solidifies.
6. Press the upper microscope slide down and wait until the agarose has solidified (at least for 1 min).
7. Carefully remove the cover slides and "slide" one microscope slide off the agarose pad.
8. Divide the pad into at least two equal sized pieces. Keep the slide in a closed 50 ml falcon tube with slightly wet tissues for further use (to prevent the thin layer of agarose drying out).
9. Each student transfers 1.5 ml of the cultures per each (both parental and mutant strains) from the culture flasks provided by the tutors into an Eppendorf tube, transfer 500ul of it and dilute in a cuvette containing 500 µl LB (1:2 dilution). Measure the OD_{600} and calculate final OD_{600} (should be between 0.8 – 1.5 to have highest T6SS activity).
10. Harvest the rest of 1 ml of your *A. baylyi* mutant and the parental strain, as well as 1 ml of *E. coli* culture (provided by the tutors), pellet (full speed, 2 min) and discard the supernatant, and resuspend each pellet in 30 µl LB each (try to avoid air bubbles while pipetting).
11. Prepare two different mixes, one containing wild type *A. baylyi* and *E. coli*, and another one with your mutant *A. baylyi* and *E. coli*, with a 2:1 *A.baylyi*:*E. coli* ratio (for example, 4 µl of *A. baylyi* and 2 µl of *E. coli*. Be sure to mix them well!
12. Spot 3 µl of each strain mix on an agarose pad and put a coverslip on top (avoid bubble formation).
13. Protect the slide from light and incubate them for at least 1 h at room temperature. Once the incubation is over, proceed to the fluorescence microscopes, where the supervisors will show you how to operate the microscopes and interpret your results. You will use

phase contrast, green fluorescent channel (VipA) and red fluorescence channel (ClpV and propidium iodide) to visualize the T6SS activities and *E. coli* killing for both your mutant and the parental strain. You will do this on a microscope **equipped with a camera** to acquire images of both your mutant and the parental strain.

9.3.2. T6SS assemblies in *A. baylyi*

14. Proceed to the fluorescence microscopes upstairs **according to your assigned time slot** and use phase contrast, green fluorescent channel (VipA) and red fluorescent channel (ClpV) to visualize T6SS activities for both your mutant and the parental strain. You will do this on a state-of-the-art microscope **equipped with a camera** to acquire images of both your mutant and the parental strain.
15. Follow the same method described in the previous section to prepare agarose pads. This time, use pads with 1% agarose in LB-PBS (no need for propidium iodide!).
16. Using your harvested *A. baylyi* cultures (see previous section), spot 3 µl of each strain on the agarose pad and put a cover slide on top (avoid bubble formation).
17. On the microscope, set up your sample, find the focus plane and choose a site that is covered 25-75% by cells. A supervisor will assist you with the camera settings.
18. Save the images for further processing in ImageJ/Fiji. Compare the T6SS dynamics of your mutant to wild-type parental strain. Take notes and put the images in your report.

9.4. Image Analysis with ImageJ/Fiji

In this session, by work with your own and provided microscopy videos, you will explore the functions of different proteins by analyzing T6SS activities quantitatively (your own videos for WT and your mutant and the provided Supplementary Videos 1-6), as well as analyzing how efficient the corresponding mutants kill *E. coli* prey visualized by live-cell imaging. You will also explore the modes of action of different T6SS effectors by analyzing provided videos how *A. baylyi* strains that contain only a single effector kill *E. coli* target cells (Supplementary Videos 7-9 under folder ADP1-single-effectors).

9.4.1. Editing images in ImageJ/Fiji

Open Fiji.

To open an image: Click on “File” – “Open”, or press CTRL+O, and open the composite file from the folder DH22_wt.

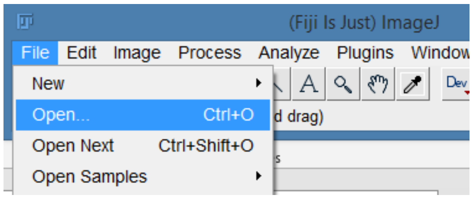

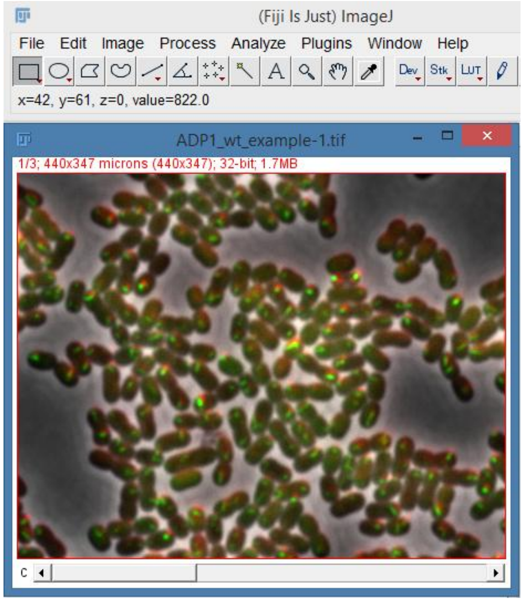

Since there is no “undo” option in Fiji it is wise to duplicate the image first. To do so click on “Image” - “Duplicate” or Ctrl+Shift+D.

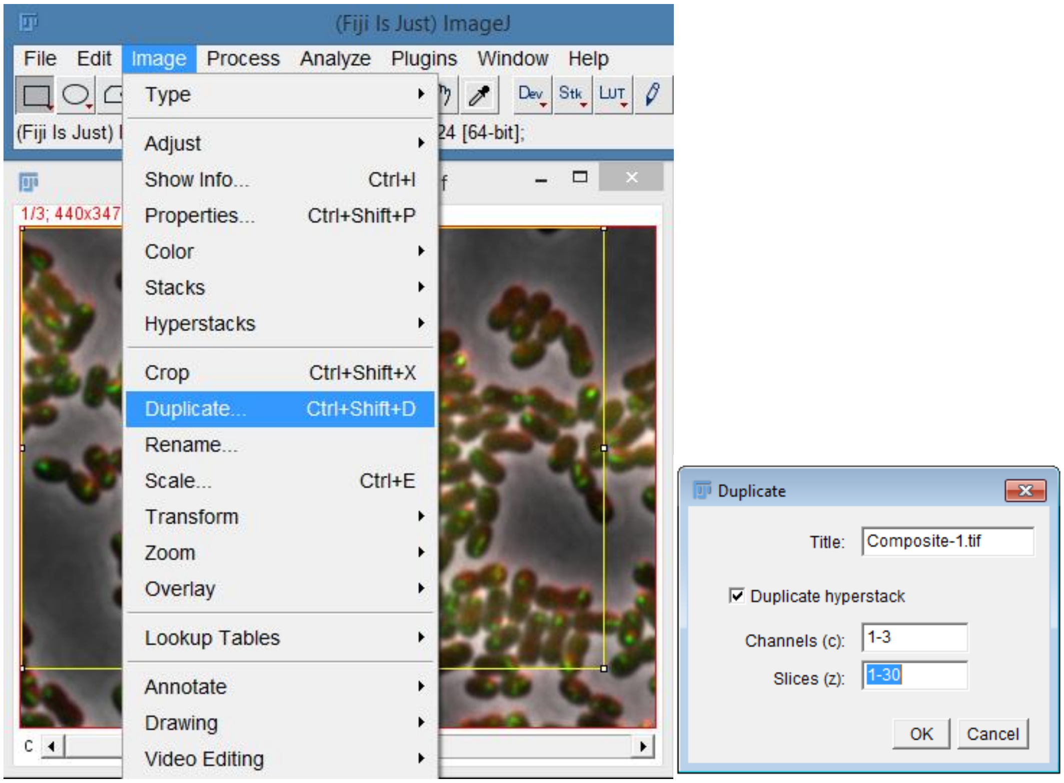

You can select the number of channels as well as the number of slices you want to duplicate. Provide a new name and confirm.

Images can have different formats (e.g. 8-bit, 16-bit… RGB) depending on the output format of the microscope software. In order to work with images in Fiji, they need to be converted to 16-bit grey scale.

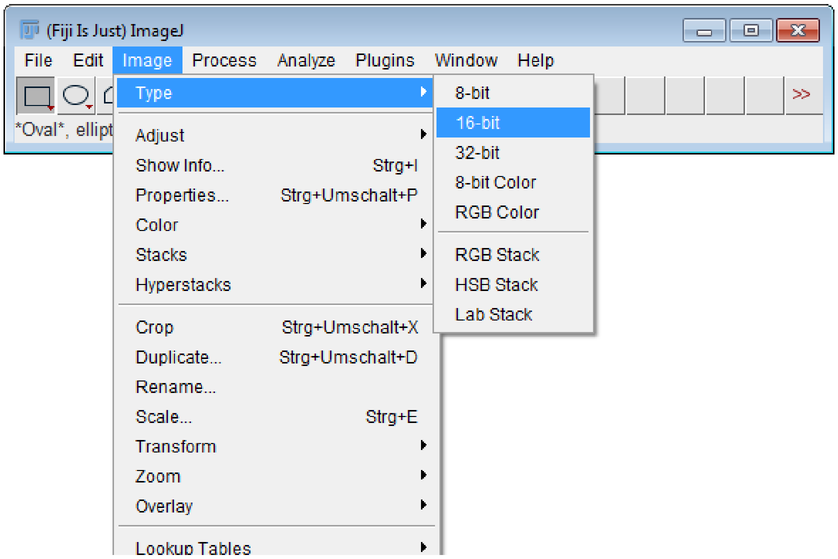

Often it is necessary to view individual channels of composite images, therefore use the "Channel Tool".

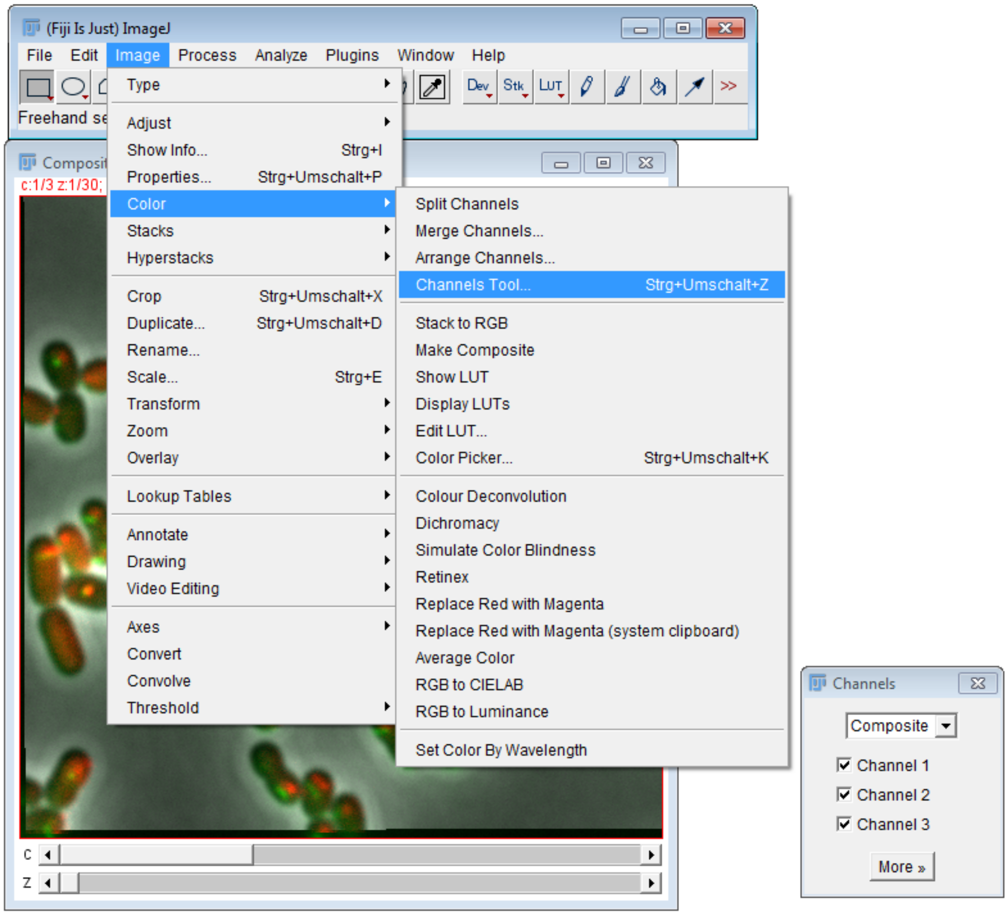

By selecting on the drop-down menu you can display your image either as a composite, a single color image or as a gray scale image. Furthermore, by clicking on the individual channels you can select and display individual channels. By clicking on "More" you can specify the display color of your selected channel.

Often it is necessary to adjust brightness and contrast for the individual channels. To do so display your image as an individual color picture, then go to "Image" - "Adjust" - "Brightness/Contrast" or press Ctrl+Shift+C.

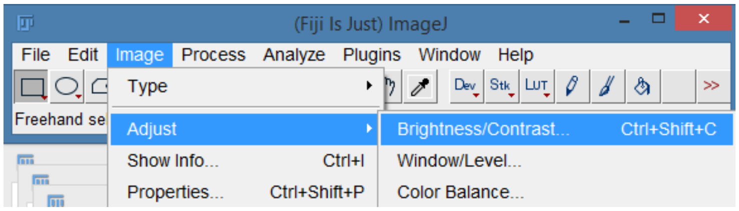

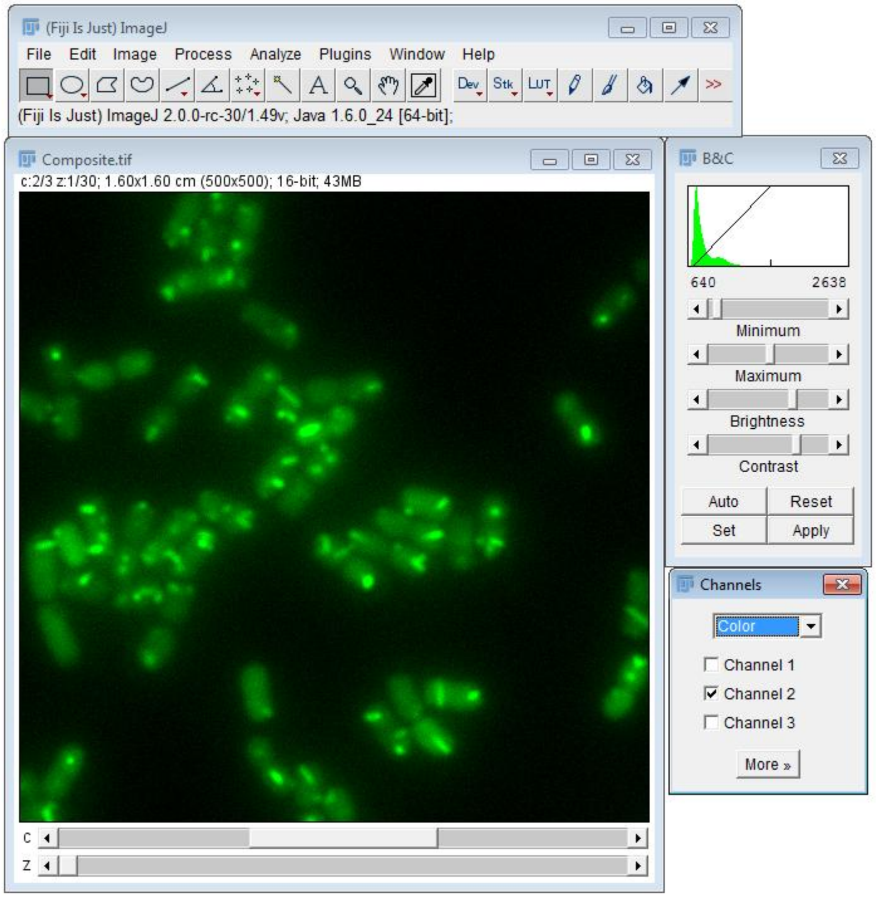

In order to merge individual images into a multi-channel composite go to "Image" – "Color" – "Merge Channels". Select the appropriate image for each channel and make sure that the text box "Create Composite" is checked.

Alternatively, you can split a composite image into individual images by selecting "Image" – "Color" – "Split Channels"

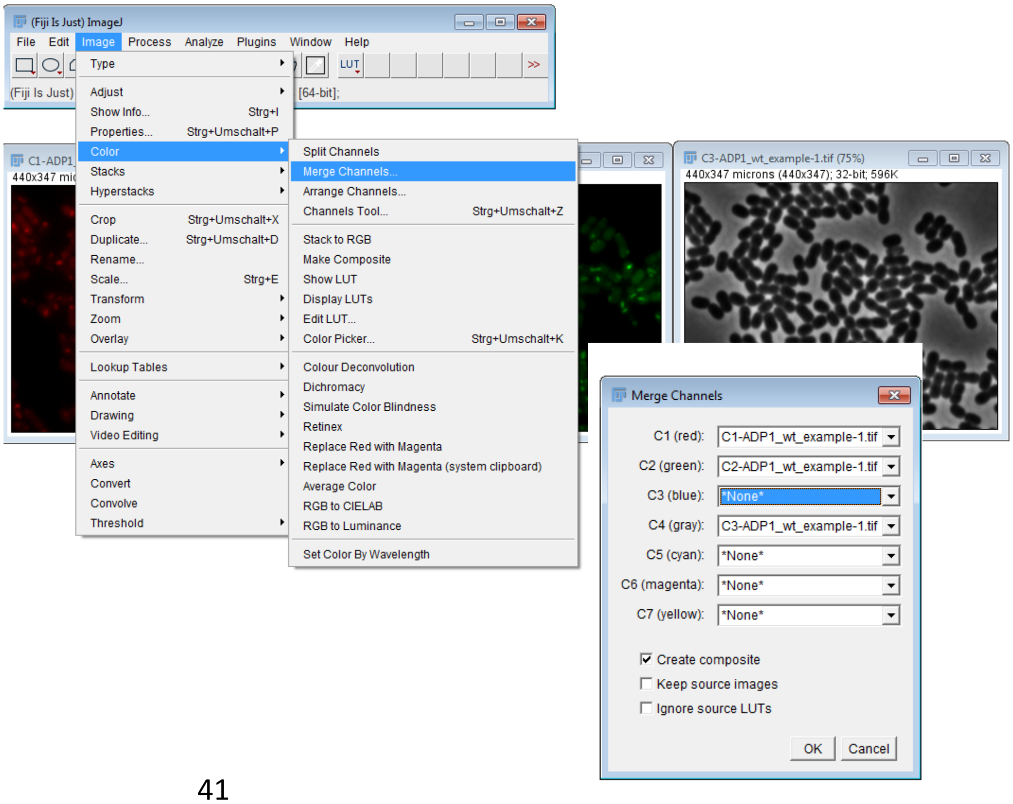

In order to save your images and to open them in Word or PowerPoint you need to convert your images back to RGB format. Go to "Image" – "Type" –"RGB". Save your files as .tiff or .jpeg. Go to "File" –"Save as" and select accordingly.

To start an animation of the stack go to "Image" – "Stacks" – "Tools" – "Start Animation" or press AltGr+\. Alternatively, you can just use the "Z-slider".

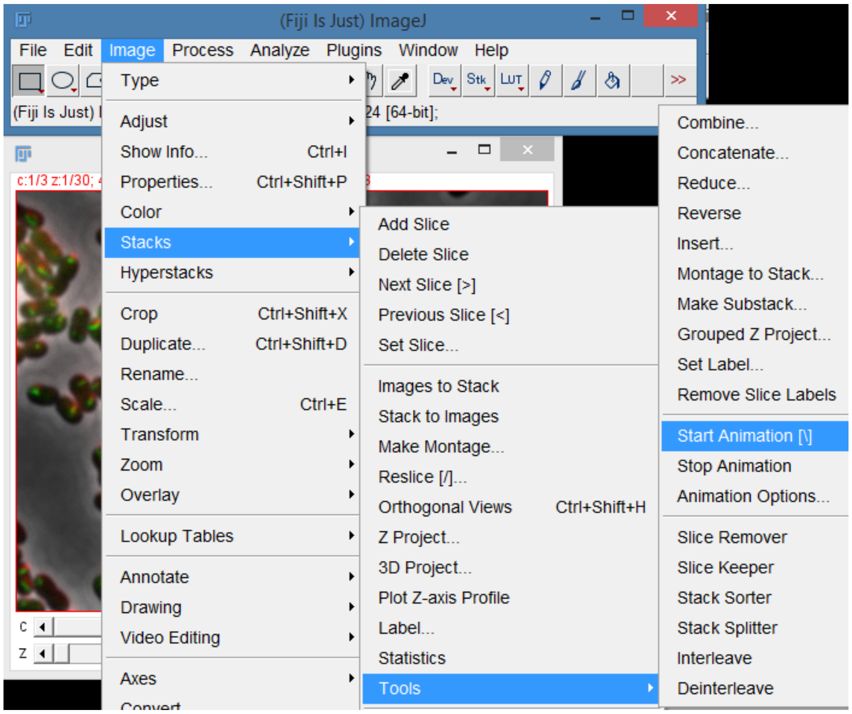

There are several options to count events in Fiji. We are going to focus just on manual counting. Change the "Point Tool" to "Multi-point Tool".

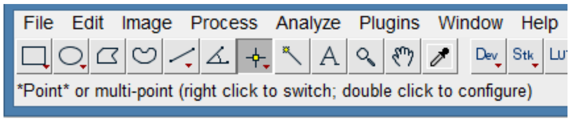

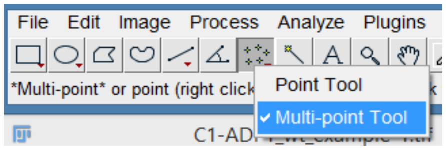

Click on events that you want to quantify.

9.4.2. Analysis of T6SS dynamics in *A. baylyi*

Now you should be familiar with the most basic features of Fiji and you can start analyzing T6SS dynamics of your *A. baylyi* mutant and compare it to the wild-type using your own videos.

- Count the number of sheath structures from the first and last slice (GFP channel) of your mutant and wild-type.
- Count the total number of *A. baylyi* cells from the GFP or mCherry (when available) images.
- What are the differences in the dynamics of VipA (GFP) and ClpV (mCherry)?
- What could be the reason for the different signal intensities in the GFP channel?
- Summarize differences between wild-type and your mutant.

9.4.3. Analysis of *E. coli* killing by *A. baylyi*

Open the provided files of wild-type and your mutant *A. baylyi* and *E. coli* mixture. When acquiring these videos, the agarose pads contain a DNA stain (SYTOX blue was used to detect loss of cell membrane integrity) allowing the detection of dead cells (cells turning blue indicating the cell death followed by the loss of cell membrane integrity).

- Count number of *A. baylyi* cells and viable *E. coli* cells from the first and the last slice.
- Compare killing efficiency of your mutant to wild-type.
- Describe the phenotypes of dying *E. coli*.

9.4.4. Analysis of single effector *A. baylyi* strains

Open all provided files of ACIAD0168, ACIAD3114 and ACIAD3425 simultaneously (Supplementary Videos 7-9). Again, a DNA stain SYTOX blue was used to detect loss of cell membrane integrity.

- Identify phenotypic differences of dying *E. coli* killed by different *A. baylyi* single effector strains.
- What could be the mode of action of each effector?

Fill out the table for your mutant and share your results with other groups so that every group can complete the table and you get a complete overview of all *A. baylyi* mutants created. How does your mutant compare to others?

<u>Talk</u> to your colleagues! The goal is to initiate discussion! (We know that all of you can read and write).

	Red-gal plate [+/-]	x-gal plate [+/-]	Quant. Killing [+/-]	Hcp secretion [+/-]	assembly [+/-]
**ACIAD0168 tae1**					
**ACIAD2685 unknown**					
**ACIAD2689 hcp**					
**ACIAD2693 tslA**					
**ACIAD2699 tagX**					
**ACIAD3425 tle1**					

‘+++’ like T6SS+; ‘++’ attenuated; ‘+’ detectable phenotype; ‘-‘ like T6SS-

10.Appendix

10.1. Bacterial strains

Organism	Genotype	Resistance
A. baylyi* ADP1 *ΔACIAD2685	ACIAD2685::*rpsL*-Kan^R cassette, ACIAD2694-mCherry2, ACIAD2691-sfGFP	Kanamycin
A. baylyi* ADP1 *ΔACIAD2685	ΔACIAD2685, ACIAD2694-mCherry2,ACIAD2691-sfGFP	Streptomycin
A. baylyi* ADP1 *ΔACIAD2689	ACIAD2689::*rpsL*-Kan^R cassette, ACIAD2694-mCherry2, ACIAD2691-sfGFP	Kanamycin
A. baylyi* ADP1 *ΔACIAD2689	ΔACIAD2689, ACIAD2691-sfGFP,ACIAD2694-mCherry2	Streptomycin
A. baylyi* ADP1 *ΔACIAD2693	ACIAD2693::*rpsL*-Kan^R cassette, ACIAD2694-mCherry2, ACIAD2691-sfGFP	Kanamycin
A. baylyi* ADP1 *ΔACIAD2693	ΔACIAD2693, ACIAD2691-sfGFP,ACIAD2694-mCherry2	Streptomycin
A. baylyi* ADP1 *ΔACIAD2699	ACIAD2699::*rpsL*-Kan^R cassette, ACIAD2694-mCherry2, ACIAD2691-sfGFP	Kanamycin
A. baylyi* ADP1 *ΔACIAD2699	ΔACIAD2699, ACIAD2694-mCherry2,ACIAD2691-sfGFP	Streptomycin
A. baylyi* ADP1 *ΔACIAD3425	ACIAD3425::*rpsL'*-Kan^R cassette, ACIAD2694-mCherry2, ACIAD2691-sfGFP	Kanamycin
A. baylyi* ADP1 *ΔACIAD3425	ΔACIAD3425, ACIAD2694-mCherry2,ACIAD2691-sfGFP	Streptomycin
***A. baylyi* ADP1 (dual color, parental, T6SS$^+$)**	ACIAD2694-mCherry2, ACIAD2691-sfGFP	Streptomycin
***A. baylyi* ADP1 (control,T6SS$^-$)**	*Δhcp* ACIAD2694-mCherry2, ACIAD2691-sfGFP	Streptomycin
***E. coli* MG1655**	wt, *lacZ*$^+$	Gentamycin

Note: ACIAD2694 is ClpV and ACIAD2691 is VipA.

10.2. Safety

Acetone:

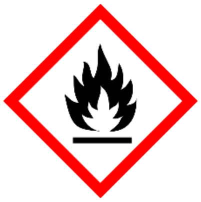 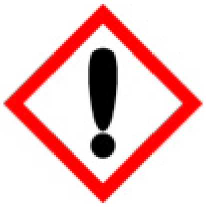

Hazards:

H225 Highly flammable liquid and vapour.
H319 Causes serious eye irritation.
H336 May cause drowsiness or dizziness.

Precautions:

P210	Keep away from heat, hot surfaces, sparks, open flames and other ignition sources. No smoking.
P261	Avoid breathing vapours.
P305 + P351 + P338	IF IN EYES: Rinse cautiously with water for several minutes. Remove contact lenses, if present and easy to do. Continue rinsing.

30 % Acrylamide-Bis solution:

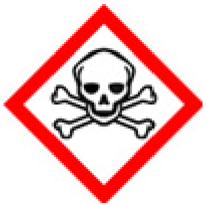 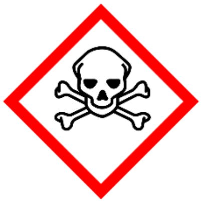

Hazards:

H301	Toxic if swallowed.
H312	Harmful in contact with skin.
H315	Causes skin irritation.
H317	May cause an allergic skin reaction.
H319	Causes serious eye irritation.
H332	May cause allergy or asthma symptoms or breathing difficulties if inhaled.
H340	May cause genetic defects.
H350	May cause cancer.
H361	Suspected of damaging fertility.
H372	Causes damage to organs through prolonged or repeated exposure.

Precautions:

P201	Obtain special instructions before use.
P280	Wear protective gloves.
P301+310	IF SWALLOWED: Immediately call a POISON CENTER/doctor.
P305 + P351 + P338	IF IN EYES: Rinse cautiously with water for several minutes. Remove contact lenses, if present and easy to do. Continue rinsing.
P308 + P313	IF exposed or concerned: Get medical advice/ attention.

APS:

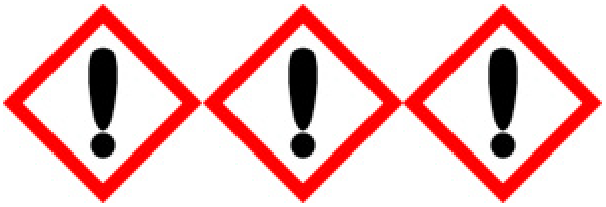

Hazards:

H272 May intensify fire; oxidiser.
H302 Harmful if swallowed.
H315 Causes skin irritation.
H317 May cause an allergic skin reaction.
H319 Causes serious eye irritation.
H334 May cause allergy or asthma symptoms or breathing difficulties if inhaled.
H335 May cause respiratory irritation.

Precautions:

P220	Keep/Store away from clothing/ combustible materials.
P261	Avoid breathing dust.
P280	Wear protective gloves.
P305 + P351 + P338 IF IN EYES:	Rinse cautiously with water for several minutes. Remove contact lenses, if present and easy to do. Continue rinsing.
P342 + P311	If experiencing respiratory symptoms: Call a POISON CENTER or doctor/physician.

Bis-Tris:

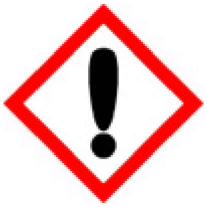

Hazards:

H315 Causes skin irritation.
H319 Causes serious eye irritation.

H335 May cause respiratory irritation.

Precautions:

P261	Avoid breathing dust/ fume/ gas/ mist/ vapours/ spray.
P305 + P351 + P338	IF IN EYES: Rinse cautiously with water for several minutes. Remove contact lenses, if present and easy to do. Continue rinsing.

DTT:

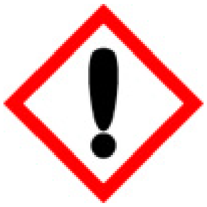

Hazards:

H302 Harmful if swallowed.
H315 Causes skin irritation.
H319 Causes serious eye irritation.
H412 Harmful to aquatic life with long-lasting effects

Precautions:

P264	Wash … thoroughly after handling
P270	Do not eat, drink or smoke when using this product.
P273	Avoid release to the environment.
P280	Wear protective gloves/protective clothing/eye protection/face protection.
P337 + P313	If eye irritation persists: Get medical advice/attention.
P501	Dispose of contents/container

Ethanol

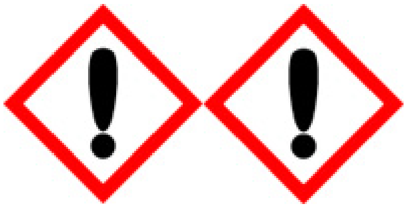

Hazards:

H225 Highly Flammable liquid and vapour
H319 Causes serious eye irritation

Precautions:

P210	Keep away from heat/sparks/open flames/hot surfaces. — No smoking.
P305+P351+P338	IF IN EYES: Rinse cautiously with water for several minutes. Remove contact lenses, if present and easy to do. Continue rinsing.

P370+P378 In case of fire: Use fire extinguisher for extinction
P403+P235 Store in a well-ventilated place. Keep cool

<u>Gentamicin:</u>

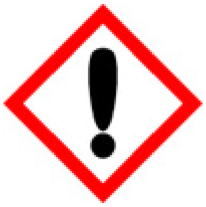

Hazards:
H317 May cause an allergic skin reaction.
H334 May cause allergy or asthma symptoms or breathing difficulties if inhaled.
Precautions:
P261 Avoid breathing dust.
P280 Wear protective gloves.
P342 + P311 If experiencing respiratory symptoms: Call a POISON CENTER or doctor/ physician.

<u>InstantBlue protein staining solution:</u>

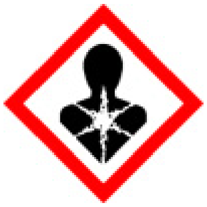

Hazards:
H290 May be corrosive to metals.
H315 Causes skin irritation.
H319 Causes serious eye irritation.
Precautions:
P305 + P351 + P338 IF IN EYES: Rinse cautiously with water for several minutes. Remove contact lenses, if present and easy to do. Continue rinsing.

<u>Kanamycin:</u>

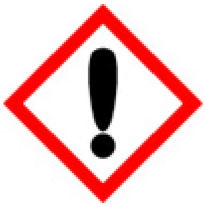

Hazards:

H360 May damage fertility or the unborn child.

Precautions:

P201 Obtain special instructions before use.

P308 + P313 IF exposed or concerned: Get medical advice/ attention

MES-running buffer:

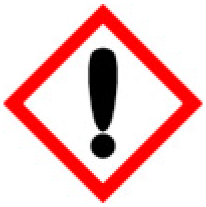

Hazards:

H316 Causes mild skin irritation.

H318 Causes serious eye damage.

Precautions:

P280 Wear protective gloves/ eye protection/ face protection.

P332+P313 If skin irritation occurs: Get medical advice/attention

NuPAGE Antioxidant solution:

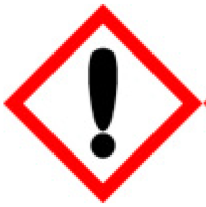
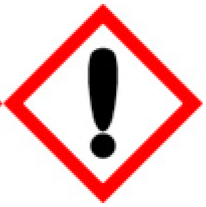

Hazards:

H319 Causes serious eye irritation

H360 May damage fertility or the unborn child

Precautions:

P201 Obtain special instructions before use

P202 Do not handle until all safety precautions have been read and understood

P264 Wash hands thoroughly after handlingP280

P280 Wear protective gloves/protective clothing/eye protection/face protection

P305 + P351 + P338 IF IN EYES: Rinse cautiously with water for several minutes. Remove contact lenses, if present and easy to do. Continue rinsing.

P337 + P313 If eye irritation persists: Get medical advice/attention.

P308 + P313 IF exposed or concerned: Get medical advice/attention.

Propidium Iodide:

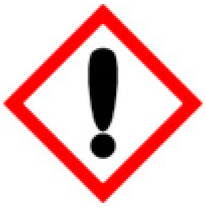

Hazards:

H315: Causes skin irritation.
H319: Causes serious eye irritation.
H335: May cause respiratory irritation.

Precautions

P264:	Wash hands thoroughly after handling.
P280:	Wear protective gloves/protective clothing/eye protection/face protection.
P362+364:	Take off contaminated clothing and wash it before reuse.
P261:	Avoid breathing dust/fume/gas/mist/vapours/spray.
P302+352:	IF ON SKIN: Wash with plenty of soap and water.
P332+313:	If skin irritation occurs, get medical advice/attention.
P305+351+338:	IF IN EYES: Rinse cautiously with water for several minutes. Remove contact lenses, if present and easy to do. Continue rinsing.
P337+313:	If eye irritation persists, get medical advice/attention.
P309+311:	Call a POISON CENTER or doctor/physician if exposed or you feel unwell.
P304+340:	IF INHALED: Remove victim to fresh air and keep at rest in a position comfortable for breathing.

SeeBlue Plus 2:

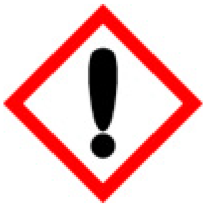

Hazards:

H351 Suspected of causing cancer if swallowed.
H360 May damage fertility or the unborn child.
H373 May cause damage to organs through prolonged or repeated exposure.

Precautions:

P201	Obtain special instructions before use
P202	Do not handle until all safety precautions have been read and understood

P260 Do not breathe dust/fume/gas/mist/vapours/spray
P280 Wear protective gloves/protective clothing/eye protection/face protection
P308 + P313 IF exposed or concerned: Get medical advice/attention
P314 Get medical advice/attention if you feel unwell

Streptomycin:

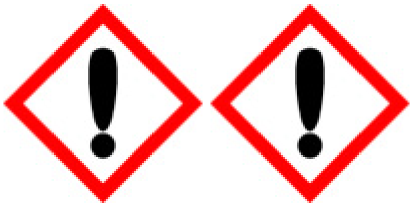

Hazards:
H302 Harmful if swallowed.
H361 Suspected of damaging fertility or the unborn child.
Precautions:
P281 Use personal protective equipment as required.

TCA:

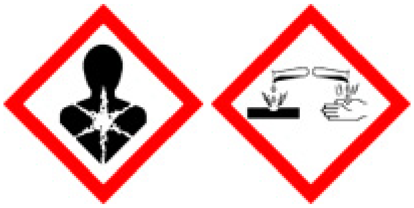

Hazards:
H314 Causes severe skin burns and eye damage.
H410 Very toxic to aquatic life with long lasting effects.
Precautions:
P260 Do not breathe dust/fumes/gas/mist/vapours/spray.
P280 Wear protective gloves/ protective clothing/ eye protection/ face protection.
P303 + P361 + P353 IF ON SKIN (or hair): Take off immediately all contaminated clothing. Rinse skin with water/shower.
P304 + P340 + P310 IF INHALED: Remove person to fresh air and keep comfortable for breathing. Immediately call a POISON CENTER or doctor/ physician.
P305 + P351 + P338 IF IN EYES: Rinse cautiously with water for several minutes. Remove contact lenses, if present and easy to do. Continue rinsing.

TEMED:

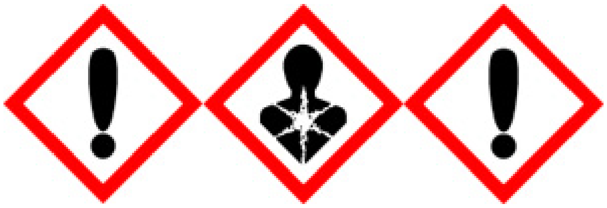

Hazards:

H225 Highly flammable liquid and vapour.
H302 Harmful if swallowed.
H314 Causes severe skin burns and eye damage.
H332 Harmful if inhaled.

Precautions:

P210	Keep away from heat/sparks/open flames/hot surfaces. - No smoking.
P280	Wear protective gloves/ protective clothing/ eye protection/ face protection.
P305 + P351 + P338	IF IN EYES: Rinse cautiously with water for several minutes. Remove contact lenses, if present and easy to do. Continue rinsing.
P310	Immediately call a POISON CENTER or doctor/ physician.

11.References

1. Desvaux, M. *et al.* (2009) Secretion and subcellular localizations of bacterial proteins: a semantic awareness issue. *Trends Microbiol* 17, 139–145

2. Cornelis, G.R. (2002) Yersinia type III secretion: send in the effectors. *J Cell Biol* 158, 401–408

3. Basler, M. *et al.* (2012) Type VI secretion requires a dynamic contractile phage tail-like structure. *Nature 2012 483:7388* 483, 182–186

4. Shneider, M.M. *et al.* (2013) PAAR-repeat proteins sharpen and diversify the type VI secretion system spike. *Nature* 500, 350–353

5. Leiman, P.G. *et al.* (2009) Type VI secretion apparatus and phage tail-associated protein complexes share a common evolutionary origin. *Proc Natl Acad Sci U S A* 106, 4154–4159

6. Young, D.M. *et al.* (2005) Opportunities for genetic investigation afforded by Acinetobacter baylyi, a nutritionally versatile bacterial species that is highly competent for natural transformation. *Annu Rev Microbiol* 59, 519–551

7. Barbe, V. *et al.* (2004) Unique features revealed by the genome sequence of Acinetobacter sp. ADP1, a versatile and naturally transformation competent bacterium. *Nucleic Acids Res* 32, 5766–5779

8. Metzgar, D. *et al.* (2004) Acinetobacter sp. ADP1: an ideal model organism for genetic analysis and genome engineering. *Nucleic Acids Res* 32, 5780

9. Basler, M. *et al.* (2013) Tit-for-tat: Type VI secretion system counterattack during bacterial cell-cell interactions. *Cell* 152, 884–894

10. Ho, B.T. *et al.* (2014) A view to a kill: the bacterial type VI secretion system. *Cell Host Microbe* 15, 9–21

11. Silverman, J.M. *et al.* (2011) Separate inputs modulate phosphorylation-dependent and -independent type VI secretion activation. *Mol Microbiol* 82, 1277–1290

12. Söding, J. *et al.* (2005) The HHpred interactive server for protein homology detection and structure prediction. *Nucleic Acids Res* 33

13. Ringel, P.D. *et al.* (2017) The Role of Type VI Secretion System Effectors in Target Cell Lysis and Subsequent Horizontal Gene Transfer. *Cell Rep* 21, 3927–3940

14. Sharma, D. *et al.* (2007) Mutational analysis of S12 protein and implications for the accuracy of decoding by the ribosome. *J Mol Biol* 374, 1065–1076

15. Rajalingam, D. *et al.* (2009) Trichloroacetic acid-induced protein precipitation involves the reversible association of a stable partially structured intermediate. *Protein Sci* 18, 980–993
