# Supplementary material for: Type VI secretion system-mediated bacterial antagonism in the classroom
Source: Access Microbiol. 2026 Jun 12;8(6):001128.v3. doi: 10.1099/acmi.0.001128.v3 (PMC13274762; doi:10.1099/acmi.0.001128.v3)
Supplement: Uncited Supplementary Material 2. [file acmi-8-01128-s002.pdf]

Sample results for experiments 3-6

Experiment 3: chromosomal knockout in *A. baylyi* (colony PCR + DNA gel)

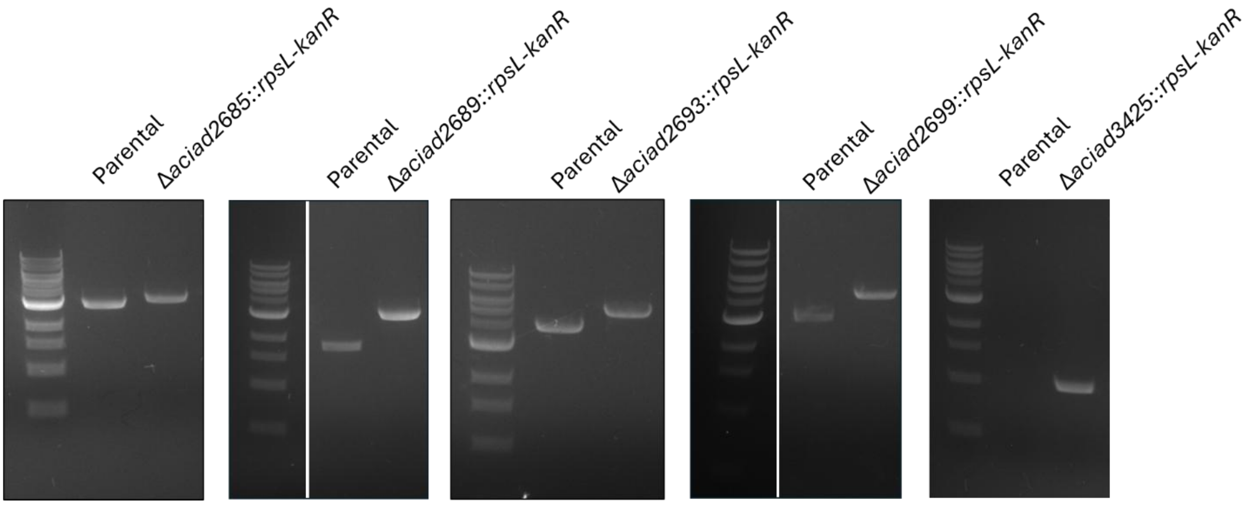

Experiments 4-6: bacterial competition assay, Hcp secretion and microscopy

Strain	Killing of *E.coli*	Hcp secretion	Structure assembly
ΔACIAD2685	None	Little to none	Very few
ΔACIAD2689	None	None	None
ΔACIAD2693	Yes, but less than WT	Yes, but less than WT	Half than WT
ΔACIAD2699	None	Little to none	Very few
ΔACIAD3425	Yes	Yes	Yes

CPRG plate

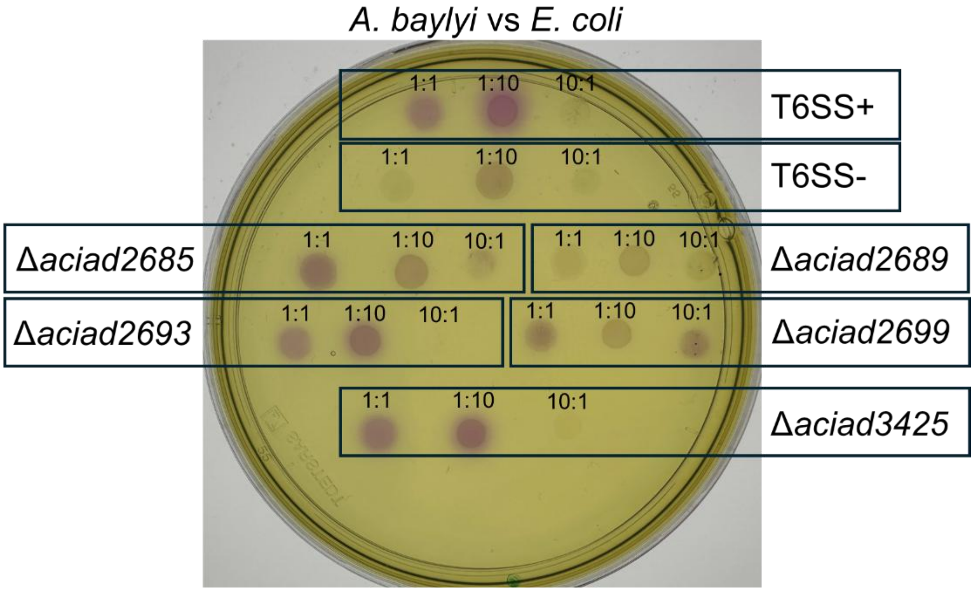

XGal plate

A. baylyi vs *E. coli*

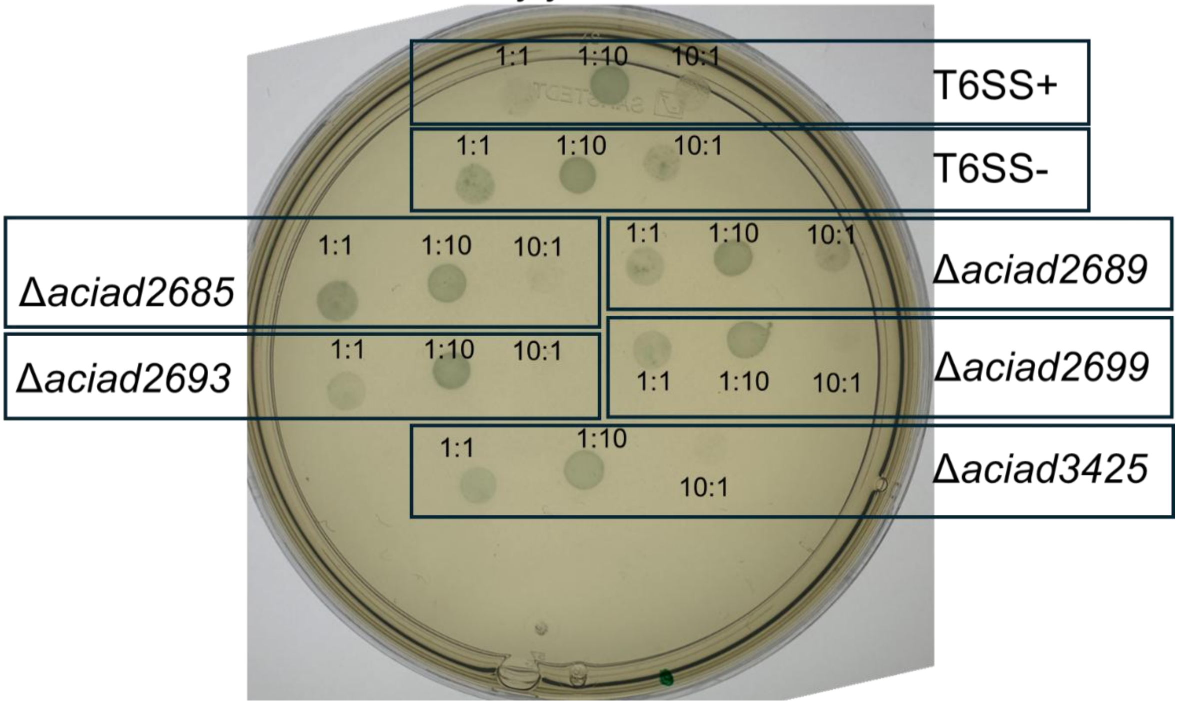

E. coli prey survival (LB + gentamicin plate) versus

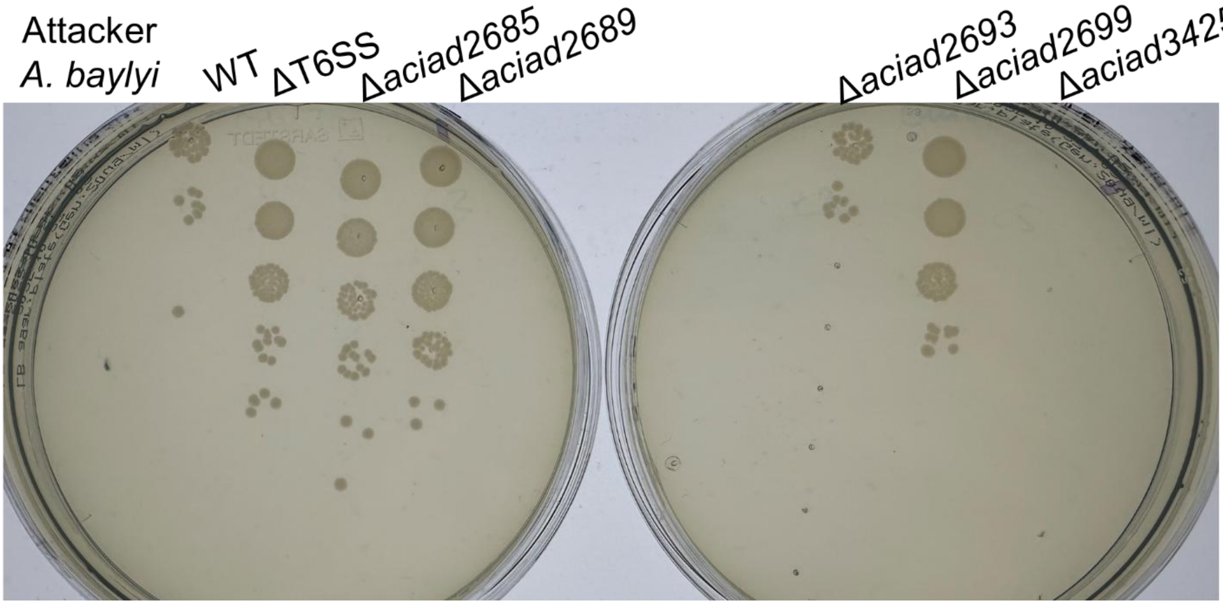

A. baylyi attacker survival (LB+streptomycin plate)

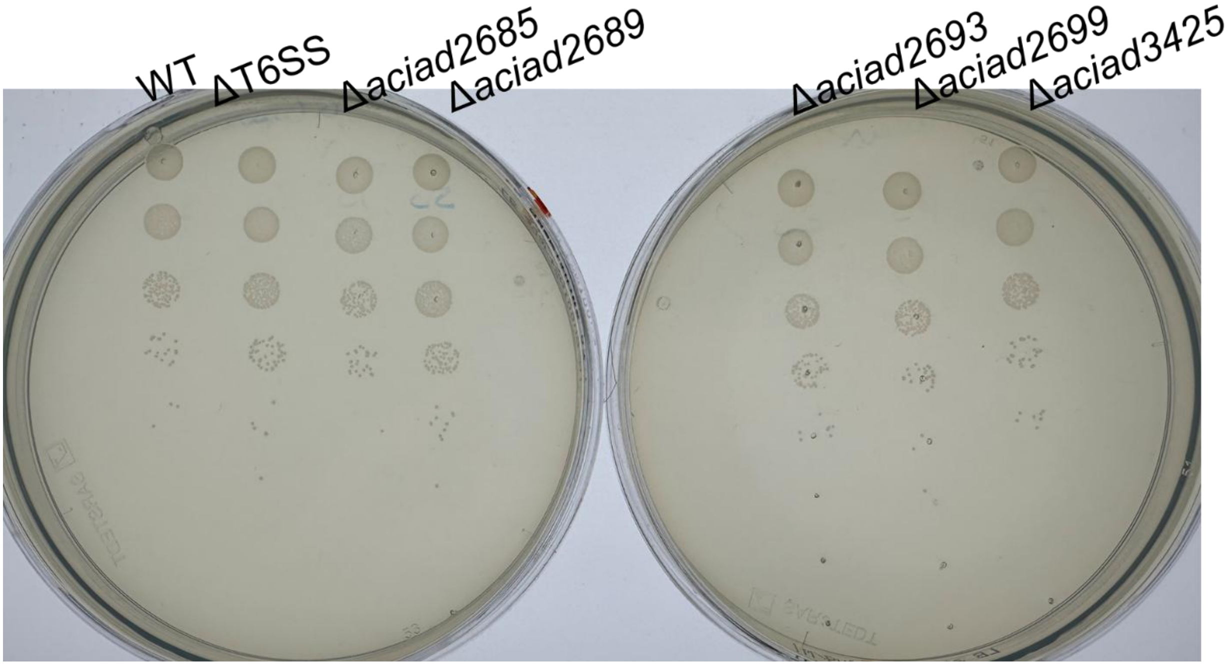

Fluorescence microscopy

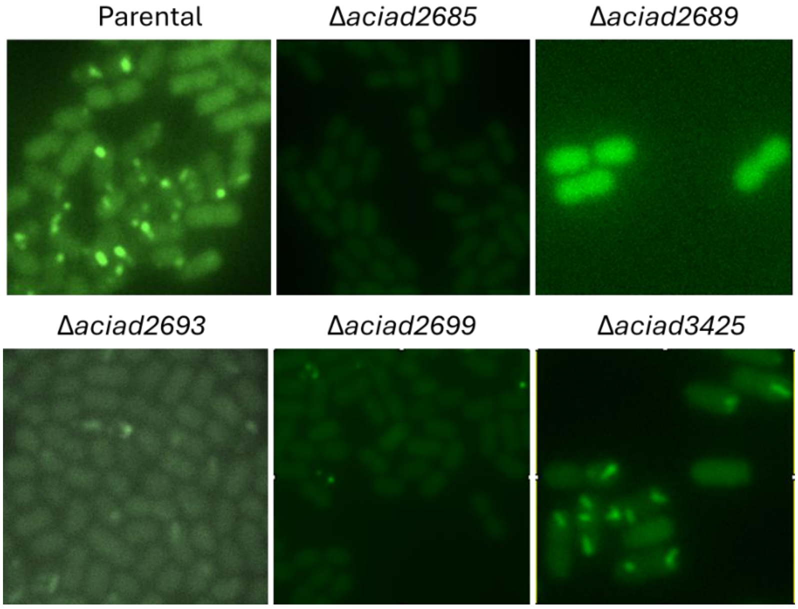

Function of the studied genes

1. *aciad2685*, unknown protein
   It is a putative membrane protein, TagY. Deletion results in nearly abolishing the assembly.

2. *aciad2689*, Hcp
   Core component of the T6SS apparatus, so called "tube-like" protein. Mutating this gene will result in no assembly at all, and no lysis/killing in any of the assays.

3. *aciad2693*, TslA
   TslA is required for contact-dependent T6SS assembly. Deletion results in a 50% reduction in assembly rate and abolishes contact-dependent assemblies. *E. coli* is still killed by this mutant, but less than by the wt strain.

4. *aciad2699*, putative zinc D-Ala-D-Ala carboxypeptidase, TagX
   According to protein blast, this gene may encode a zinc D-Ala-D-Ala carboxypeptidase. A deletion strain does not kill *E. coli* as expected. In fluorescent microscopy, only a few structures wandering around can be observed, so this putative peptidase may be one key component in assembly.

5. *aciad3425*, putative lipase, Tle1
   Secreted peptides from this protein are detected only in the T6SS positive ADP1 strain, so it is a secreted T6SS effector. Blast of this protein indicates that it is a putative lipase. Deletion has no effect on assembly or *E. coli* killing. *E. coli* cells shrink and then fade away while becoming blue.
