# Supplementary material for: Type VI secretion system-mediated bacterial antagonism in the classroom
Source: Access Microbiol. 2026 Jun 12;8(6):001128.v3. doi: 10.1099/acmi.0.001128.v3 (PMC13274762; doi:10.1099/acmi.0.001128.v3)
Supplement: Uncited Supplementary Material 3. [file acmi-8-01128-s003.pdf]

Supplementary Table 1. Primers used for the verification of the insertion of the counterselectable cassette in activity 2.

Primer	Sequence	T_M [°C]	Annealing temp. [°C]	PCR product WT/mutant [bp]
**ACIAD2685_Det6_For**	cgcgcagatttttggcaagt	60	61/±3°C	2943/3157
**ACIAD2685_Det6_Rev**	gcgatgagcggattgagatg	58		
**ACIAD2689_Det6_For**	tcgattcgggctgcaattaa	55	58/±3°C	1979/3271
**ACIAD2689_Det6_Rev**	agaggatgtgactggtcagga	59		
**ACIAD2693_Det6_For**	ggtcaatgcgacataacgtg	58	57/±3°C	3211/4222
**ACIAD2693_Det6_Rev**	ttgaccacctgcaccaatta	54		
**ACIAD2699_Det6_For**	tttctgcacctgccgaagaa	57	58/±3°C	2349/3234
**ACIAD2699_Det6_Rev**	acccatggtcaagtacagga	55		
**ACIAD3425_Det_For**	ggcctgagtgtcgtattgct	59	58/±3°C	-/1022
**PrpsL-R**	atggcaattctcccgttaat	55		

Note: Students working on ACIAD3425 will need special primers, since their PCR product has the exact same length as wild-type. Therefore, they will use a primer binding to the cassette and a detection primer at the corresponding locus in the genome. There won't be any PCR product in the control.
